# Quantification of extracellular vesicles *in vitro* and *in vivo* using sensitive bioluminescence imaging

**DOI:** 10.1080/20013078.2020.1800222

**Published:** 2020-08-21

**Authors:** Dhanu Gupta, Xiuming Liang, Svetlana Pavlova, Oscar P.B Wiklander, Giulia Corso, Ying Zhao, Osama Saher, Jeremy Bost, Antje M. Zickler, Andras Piffko, Cecile L. Maire, Franz L. Ricklefs, Oskar Gustafsson, Virginia Castilla Llorente, Manuela O. Gustafsson, R. Beklem Bostancioglu, Doste R Mamand, Daniel W. Hagey, André Görgens, Joel Z. Nordin, Samir EL Andaloussi

**Affiliations:** a Biomolecular Medicine, Clinical Research Center, Department of Laboratory Medicine, Karolinska Institutet, Stockholm, Sweden; b Experimental Cancer Medicine, Clinical Research Center, Department of Laboratory Medicine, Karolinska Institutet, Stockholm, Sweden; c Clinical Research Center, Karolinska University Hospital, Stockholm, Sweden; d Department Pharmaceutics and Industrial Pharmacy, Faculty of Pharmacy, Cairo University, Cairo, Egypt; e Department of Neurosurgery, University Medical Center Hamburg-Eppendorf, Hamburg, Germany; f Evox Therapeutics Limited, Oxford, UK; g Institute for Transfusion Medicine, University Hospital Essen, University of Duisburg-Essen, Essen, Germany; h Department of Molecular Therapy, National Institute of Neuroscience, National Center of Neurology and Psychiatry (NCNP), Tokyo, Japan; i Department of Biology, Faculty of Science, Cihan University-Erbil, Iraq

**Keywords:** Biodistribution, bioluminescence, evs Labelling, drug delivery, exosomes, extracellular vesicles, microvesicles, nanotechnology, evs subpopulation, pharmacokinetics

## Abstract

Extracellular vesicles (EVs) are naturally occurring nano-sized carriers that are secreted by cells and facilitate cell-to-cell communication by their unique ability to transfer biologically active cargo. Despite the pronounced increase in our understanding of EVs over the last decade, from disease pathophysiology to therapeutic drug delivery, improved molecular tools to track their therapeutic delivery are still needed. Unfortunately, the present catalogue of tools utilised for EV labelling lacks sensitivity or are not sufficiently specific. Here, we have explored the bioluminescent labelling of EVs using different luciferase enzymes tethered to CD63 to achieve a highly sensitive system for *in vitro* and *in vivo* tracking of EVs. Using tetraspanin fusions to either NanoLuc or ThermoLuc permits performing highly sensitive *in vivo* quantification of EVs or real-time imaging, respectively, at low cost and in a semi-high throughput manner. We find that the *in vivo* distribution pattern of EVs is determined by the route of injection, but that different EV subpopulations display differences in biodistribution patterns. By applying this technology for real-time non-invasive *in vivo* imaging of EVs, we show that their distribution to different internal organs occurs just minutes after administration.

## Graphical abstract

**Figure:**
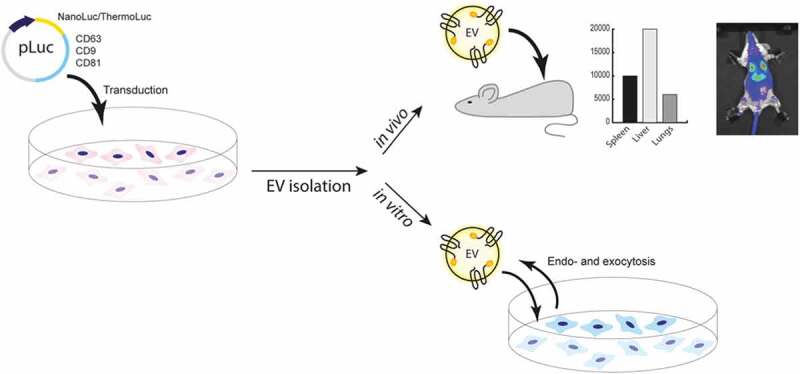

## Introduction

Extracellular vesicles (EVs) are a diverse population of lipid-enclosed particles containing an array of nucleic acid and protein macromolecules, which are secreted by all cell types and can be found in all body fluids[1]. The most common nomenclature subdivides them into three major subclasses based on their biogenesis: exosomes, which is around 40–130 nm and originates from the endolysosomal pathway; microvesicles, which bud directly from the plasma membrane and can range from 50 to 1000 nm and apoptotic bodies, which are the remnants of dying cells and are between 1 and 4 µm[1–2]. EVs are important for cell-to-cell communication and can deliver large macromolecules such as mRNA and proteins to recipient cells [3]. Recent evidence has demonstrated their importance in physiology as well as disease pathology, especially in tumour development, where they have been implicated in the spread of metastasis [4,5]. Because of their innate ability to transfer various biological effector cargoes between different cell types, EVs have attracted interest as cell-free nano-therapeutics. For instance, the anti-inflammatory and regenerative effects of mesenchymal stromal cell (MSC) derived EVs have shown therapeutic potential in various inflammatory models, including graft versus host disease in man [6–8]. Moreover, a growing number of EV engineering strategies have paved the way toward an efficient drug delivery system for diverse therapeutics, ranging from nucleic acid- and protein-based cargos to small molecules. However, in order for EVs to achieve the goal of tissue-specific delivery of therapeutically relevant effector molecules, the development of better analytical tools are necessary [9–12].

The growing interest in EVs over the last decade has led us and others to develop various EV labelling strategies, both exogenous and endogenous, to monitor the fate of EVs *in vivo* [13–22]. Recently, nuclear and magnetic resonance imaging-based approaches involving exogenous labelling of EVs with radionuclides or MRI contrast fluid have emerged [23–26]. Although these strategies provide exceptional tissue penetration and quantification, these approaches are hard to implement in basic scientific research as they are expensive and time-consuming. Another exogenous loading strategy is to utilise lipophilic fluorescent dyes such as DiR, DiI, DiD and PKH [16,18,27]. However, because of the non-covalent association of dyes with the EV surface, there is a high risk of shuttling to other cell membranes that do not reflect EV biodistribution. Importantly, no exogenous labelling strategies will reflect the half-life of EVs, as these contrast agents and dyes are highly stable and resistant to degradation [28]. Therefore, strategies involving reporter proteins loaded as cargo have been developed to better determine EV half-life [13,20,22]. Although fluorescent proteins offer a versatile system for EV labelling and detection, tissue autofluorescence has restricted the use of these proteins to *in vitro* studies. Therefore, bioluminescent strategies for EV labelling have gained increased attention. Previous reports have utilised Gaussia and Renilla luciferases for labelling of EVs and, while these enzymes are stable, they lack the dynamic range and signal intensity necessary to determine their *in vivo* distribution pattern [17,19,20,22,29,30]. More recently, others have utilised NanoLuc for EV labelling for *in vitro* applications, but *in vivo* applications of NanoLuc remain unexplored [12,31].

In this study, we addressed these issues by exploring the endogenous labelling of EVs with different luciferase reporter proteins. We performed a systematic comparison of five different luciferases with different stability and brightness properties. Furthermore, we applied this technology to explore various *in vitro* and *in vivo* applications relevant to EV research, and experimentally demonstrate the potential pitfalls associated with these systems. This study is the first to assess *in vivo* pharmacokinetic patterns of EVs by quantifying the number of EVs and show the distribution of EVs in real time directly after the injection into mice. In addition, we explore how different routes of injection, including intravenous, intraperitoneal, subcutaneous, intracardiac, intracarotid, intracranial and peroral, affect EV biodistribution. Finally, we demonstrate how different subpopulations of EVs differ in their *in vivo* biodistributions. Our findings emphasize the usefulness of this technology in the different areas of EV research, spanning from therapeutics to disease pathophysiology.

## Materials and methods

### Cell culture

HEK-293T, Huh7 and B16F10 cells were cultured in high glucose DMEM medium (Gibco, USA) supplemented with 10% FBS (Gibco, USA) and 1% Anti-anti (Invitrogen, USA) in a humidified incubator set at 37°C and 5% CO_2_. Cord blood MSCs were cultured in RPMI (Gibco, America) supplemented with 10% FBS (Gibco, USA) and 1% Antibiotic-Antimycotic (Invitrogen, USA) with the same incubator settings as above.

For EV purification, cells were plated at 60% confluence in a 15 cm dish (Corning, USA). Twenty-four hours after cell plating, the growth media was changed to Opti-MEM (Gibco, USA). Forty-eight hours later, cells reached 90% confluence, and the conditioned medium (CM) was collected for purification.

### Generation of different constructs

Codon-optimized DNA sequences coding for human CD63 (Uniprot accession number P08962), human CD81 (Uniprot accession number P60033), human CD9 (Uniprot accession number P21926) and the luciferase proteins NanoLuc (GenBank accession number AGG56535.1), ThermoLuc [32], Super Rluc8 [33] and Firefly (GenBank accession number AJD87366.1) were synthesized (Integrated DNA Technologies) as gene fragments and cloned downstream of the CAG promoter into the pLEX vector backbone using EcoRI and NotI. To generate the different constructs expressing luciferase proteins fused to the C-terminus of CD63, luciferase protein-coding sequences (CDS) were subcloned into pLEX-CD63 using SacI and NotI. For cloning the luciferase gene for N terminus CD63 fusion, luciferase protein CDS were subcloned into pLEX-CD63 using EcoRI and BsiWI. Next, the complete CDS of the different CD63-luciferase protein fusions were cloned into the lentiviral p2CL9IPwo5 backbone downstream of the SFFV promoter using EcoRI and NotI, and upstream of an internal ribosomal entry site-puromycin resistance cDNA cassette. All expression cassettes were confirmed by sequencing. Lentiviral supernatants were produced as described previously [34]. In brief, HEK-293T cells were co-transfected with p2CL9IPw5 plasmids containing CD63 fused to luminescent proteins, the helper plasmid pCD/NL-BH, and the human codon-optimized foamy virus envelope plasmid pcoPE using the transfection reagent JetPEI (Polyplus, Illkrich Cedex). 16 hours post transfection, gene expression from the human CMV immediate-early gene enhancer/promoter was induced with 10 mM sodium butyrate (Sigma-Aldrich) for 6 hours before fresh media was added to the cells, and the supernatant was collected 22 hours later. Viral particles were pelleted at 25,000 × *g* for 90 min at 4°C. The supernatant was discarded, and the pellet was re-suspended in 2 ml of Iscove’s Modified Dulbecco’s Media supplemented with 20% FBS and 1% P/S. Aliquots were stored at −80°C until usage. To generate stable cell lines, HEK-293T cells were transduced by overnight exposure to virus stocks and passaged at least five times under puromycin selection (Sigma; 6 µg/mL).

### Transfection

HEK-293T cells were seeded into 15 cm dishes, 10 million cells/dish 1 day before transfection. A total of 30 µg of plasmid was mixed with 2 ml Opti-MEM in one tube for 5 min at room temperature, and 45 µg of Polyethylenimine (PEI) (Sigma) was mixed with 2 ml Opti-MEM in another tube with the same incubation conditions. Then, the plasmid and PEI were mixed in one tube and incubated for 20 min at room temperature. At last, the DNA-PEI mixture was added into the medium in a dropwise manner. The medium was changed to Opti-MEM with 1% Anti-anti 3–4 hours post-transfection, and EVs were harvested 48 hours after medium change.

### EV isolation

CM was pre-cleared first by a low-speed centrifugation step (500 × *g* for 10 min) followed by centrifugation at 2,000 × *g* for 10–20 min to remove larger particles and debris. Unless indicated otherwise, samples were subsequently filtered through a syringe filter (VWR) or bottle top filters (Corning, low protein binding) with cellulose acetate membrane with a 0.22 µm pore size to remove any remaining larger particles. The CM was then ultra-filtrated either using Amicon Ultra-15 100 kDa (Millipore) spin filter at 4000 x g for 30 min or using tangential flow filtration (MicroKross, 20 cm^2^, SpectrumLabs) with a cut-off of 300 kDa to concentrate the CM. In some experiments, the concentrated retentate was further purified by size exclusion chromatography column (iZON biosciences) according to the manufacturer’s instruction to further purify the EVs.

For DiR labelling, filtered CM was incubated with 1 µM fluorescent lipophilic tracer DiR (1,1-dioctadecyl-3,3,3,3-tetramethylindotricarbocyanine iodide) (D12731, Invitrogen, Life Technologies) at room temperature (RT) for 30 min prior to EV isolation by ultracentrifugation at 120,000 x g for 70 min (Beckman coulter).

### Nanoparticle tracking analysis (NTA)

Nanoparticle tracking analysis was applied to determine the particle size and concentration of all samples. All samples were characterized with a NanoSight NS500 instrument equipped with NTA 2.3 analytical software and a 488 nm laser. At least five 30 secondsvideos were recorded per sample in light scatter mode with a camera level of 11–13. Software settings for analysis were kept constant for all measurements (screen gain 10, detection threshold 7). All samples were diluted in 0.22 µm filtered PBS to an appropriate concentration before analysis.

### Western blotting

HEK-293T cells were collected and counted using trypan blue 0.4% (Invitrogen, Thermo Fisher Scientific) in a Countess II FL automated cell counter (Invitrogen, Thermo Fisher Scientific). 2×10^6^ cells were pelleted at 300 × g for 5 min, washed once with cold PBS and pelleted again at 300 × g for 5 min. The cell pellet was lysed with 100 µL of RIPA buffer, kept on ice, and vortexed five times every 5 min. The cell lysate was then spun at 12,000 × g for 10 min at 4°C and the supernatant was transferred to a new tube and kept on ice. Thirty-thousand cells or 5×10^9^ EVs were mixed with a buffer containing 0.5 M dithiothreitol, 0.4 M sodium carbonate (Na_2_CO_3_), 8% SDS, and 10% glycerol, and heated at 65°C for 5 min. The samples were loaded onto a NuPAGE Novex 4–12% Bis-Tris Protein Gel (Invitrogen, Thermo Fisher Scientific) and run at 120 V in NuPAGE MES SDS running buffer (Invitrogen, Thermo Fisher Scientific) for 2 hours. The proteins on the gel were transferred to an iBlot nitrocellulose membrane (Invitrogen, Thermo Fisher Scientific) for 7 min at 20 V using the iBlot system. The membrane was blocked with Odyssey blocking buffer (LI-COR) for 60 min at RT with gentle shaking. After blocking, the membrane was incubated overnight at 4°C or 1 hour at RT with primary antibody solution [1:1000 dilution for anti-Alix (ab117600, Abcam), anti-Tsg101 (ab30871, Abcam) and anti-NanoLuc (Promega); 1:2000 dilution for anti-CD9 (ab92726, Abcam)]. The membrane was washed with PBS supplemented with 0.1% Tween-20 (PBS-T, Sigma) five times for 5 min each and incubated with the corresponding secondary antibody (LI-COR) for 1 hour at RT (1:15,000 goat anti-mouse IRDye800CW or 680LT to detect Alix and NanoLuc; 1:15,000 dilution goat/anti-rabbit IRDye800CW or 680LT to detect CD9, Tsg101). The membrane was washed with PBS-T five times over 25 min, twice with PBS and visualized on the Odyssey infrared imaging system (LI-COR).

### Bead-based multiplex exosome flow cytometry assay

Conditioned media samples were subjected to bead-based multiplex EV analysis by flow cytometry (MACSPlex Exosome Kit, human, Miltenyi Biotec) as described previously [34]. Briefly, samples were processed as follows: EV-containing, pre-cleared CM samples (120 µl) were loaded onto wells of a pre-wet and drained MACSPlex 96-well 0.22 µm filter plate before 15 µL of MACSPlex Exosome Capture Beads (containing 39 different antibody-coated bead subsets) were added to each well. Filter plates were then incubated on an orbital shaker overnight (14–16 hours) at 450 rpm at room temperature protected from light. To wash the beads, 200 µL of MPB was added to each well, and the filter plate was put on a vacuum manifold with vacuum applied (Sigma-Aldrich, Supelco PlatePrep; −100 mBar) until all wells were drained. For counterstaining of EVs bound by capture beads with detection antibodies, 135 µL of MPB and 5 µL of each APC-conjugated anti-CD9, anti-CD63 and anti-CD81 detection antibody were added to each well, and plates were incubated on an orbital shaker at 450 rpm protected from light for 1 hours at room temperature. Next, plates were washed by adding 200 µL MPB to each well followed by draining on a vacuum manifold. This was followed by another washing step with 200 µL of MPB, incubation on an orbital shaker at 450 rpm protected from light for 15 min at room temperature and draining all wells again on a vacuum manifold. Subsequently, 150 µL of MPB was added to each well, beads were re-suspended by pipetting and transferred to V-bottom 96-well microtiter plate (Thermo Scientific). Flow cytometric analysis was performed with a MACSQuant Analyser 10 flow cytometer by using the built-in 96-well plate reader. All samples were automatically mixed immediately before 70–100 µL were loaded to and acquired by the instrument, resulting in approximately 7,000–12,000 single bead events being recorded per well. FlowJo software (v10, FlowJo LLC) was used to analyse flow cytometric data. Median fluorescence intensity (MFI) for all 39 capture bead subsets was background corrected by subtracting respective MFI values from matched non-EV buffer or media controls that were treated exactly like EV-containing samples (buffer/medium + capture beads + antibodies).

## Statistical analysis

GraphPad Prism 8 (GraphPad Prism Software, La Jolla, CA, USA) was used to analyse data and assemble figures. To generate heatmaps of data, flow cytometric data were gated in FlowJo with gated data being exported to comma-separated files, which were subsequently imported into MATLAB (v9.3.0, Mathworks Inc.) for further analysis and data visualization.

### Detection of RLU for different luciferases

For the detection of NanoLuc luciferase activity, 30 μL of purified EVs solution or CM was added into white-walled 96-well plates along with 30 μL Nano-Glo substrate diluted 1:50 in the provided buffer (Nano-Glo Luciferase Assay System: Promega), as per the manufacturer instructions. The luciferase intensity in each well was immediately measured using a GloMax® 96 Microplate Luminometer machine (Promega). For NanoLuc localization experiments, EVs or CM were incubated with 100 µg/ml Proteinase K (Qiagen) at 37°C for 3 hours prior to NanoLuc measurement.

For the detection of ThermoLuc and Firefly luciferase activity, 30 μL purified EVs solution or CM (lysed in 0.1% triton X-100) was added into white-walled 96-well plates along with 30 μL Luciferin substrate (Firefly Luciferase Assay System: Promega), as per the manufacturer instructions. The luciferase intensity in each well was immediately measured using a GloMax® 96 Microplate Luminometer machine (Promega).

For the detection of Super Rluc8, 30 μL purified EVs solution or CM (lysed in 0.1% triton X-100) was added into white-walled 96-well plates along with 30 μL 1 mg/ml Coelenterazine (Sigma) diluted 1:50 in PBS. The luciferase intensity in each well was immediately measured using a GloMax® 96 Microplate Luminometer machine (Promega).

### EV uptake by recipient cells

For comparison, HEK-293T CD63-NanoLuc derived EVs were isolated with Tangential flow filtration (TFF)/size exclusion chromatography (SEC) as described above. Particle concentration and size were analysed with NTA in scatter mode. A range of EV doses were added to human hepatocellular carcinoma (Huh7) or B16-F10 melanoma cells seeded the day before at a density of 1×10^4^ cells per well in a 96-well plate. Cells were incubated for 2 hours at 37°C, 5% CO_2_ atmosphere. After incubation, the cells were washed twice with PBS and lysed in 100 µl of Dulbecco’s PBS (Invitrogen) and 0.1% TritonX-100. The plate was then incubated on an orbital shaker at room temperature for 10 min for complete lysis of the cells. Cell lysate was then analysed for luciferase activity using the appropriate substrates as detailed above.

### EV release by producing cells

HEK-293T CD81-ThermoLuc, HEK-293T CD63-ThermoLuc and HEK-293T CD9-ThermoLuc cells were seeded at a density of 1×10^3^ cells/well in high glucose Dulbecco’s modified Eagle’s medium (DMEM) (Invitrogen) supplemented with 10% foetal bovine serum (FBS) (Invitrogen). After one day, media was removed and cells were washed thoroughly with warm PBS before adding either serum-free DMEM media, Opti-MEM® or full media (DMEM+10%FBS) and incubating the plates at 37°C in a humidified incubator with 5% CO_2_.

At assigned time points, media was collected and cells were either lysed directly using 1% Triton-X 100 in PBS for 10 min or trypsinized and collected for later lysis. For collected conditioned media, samples were centrifuged at 900 g for 5 min to get rid of debris before lysis by adding appropriate volumes of 10% Triton-X 100 to a final concentration of 1% and shaking for 10 min.

To measure luciferase signal, 30 µL of the lysates was added to white 96-well plates and mixed with 25 µL of luciferase reagent (Promega) added by an injector. The relative light units (RLU) of luciferase were determined (GloMax® 96 Microplate Luminometer machine-Promega, Sweden) with 10 Seconds integration time and 2 Seconds delay between injection and measurement. For cell lysates, 5 µL was used to determine total protein quantity using the DC Protein Assay (Bio-Rad).

### Transmission electron microscopy

Ten microlitres of EV re-suspension in PBS were added onto glow-discharged formvar-carbon-coated grids (TED Pella Inc.) for 1 min. The grids were blotted with filter paper and washed with double-distilled water, blotted dry and stained with 2% uranyl acetate (UA) for 1 min. The grids were blotted dry and left to air-dry for 15 min. EVs were imaged with an FEI Tecnai 10 transmission electron microscope at an accelerating voltage of 100 kV.

### Mouse experiments

The following experiments were performed under ethical permission granted by Swedish Jordbruksverket (Ethical Permit S4-16)

For EV *in vivo* pharmacokinetic studies, female NMRI mice with a bodyweight of around 20 g were IV (tail vein) injected with 1×10^11^ Wharton Jelly CD63-NanoLuc MSC-EVs in 100 µL PBS. Animals were sacrificed at different time points (5 min, 15 min, 30 min, 60 min, 6 hours and 24 hours), blood was sampled by heart puncture, collected into PST-tubes (BD Biosciences) and processed according to the manufacturer’s instructions. The animals were then perfused using cold PBS and different organs were harvested and stored in 2 ml Eppendorf tubes at −80°C until further use.

To study the effect of different injection routes on EV biodistribution *in vivo*. Female NMRI mice with a bodyweight of around 20 g were IV (tail vein), IP, P.O., SC injected with 1×10^11^ HEK-293T CD63-NanoLuc EVs in 100 µL PBS. Animals were blood sampled by tail vein at different time points and collected into PST-tubes (BD Biosciences) and processed according to the manufacturer’s instructions. Animals were sacrificed at 6 hours and 24 hours post injection. Different organs were harvested and stored in 2 ml Eppendorf tubes at −80°C until further use.

To measure EVs *in vivo* biodistribution with ThermoLuc EVs labelled with or without DiR, female NMRI mice with a bodyweight of around 20 g were IV (tail vein) injected with ThermoLuc EVs in 100 µL PBS. Animals were imaged by IVIS Spectrum (Perkin Elmer) for ThermoLuc luminescence after IP administration of 150 mg/kg D luciferin or DiR Fluorescence. Here, either live (isoflurane sedated) mice were imaged or the animals were sacrificed and the organs harvested prior to analysis.

For real-time imaging of the EVs *in vivo* biodistribution, female NMRI mice with a bodyweight of around 20 g were administered IP with 150 mg/kg D luciferin, and after 5 min, ThermoLuc EVs in 100 µL PBS were injected IV (tail vein). Live animals (isoflurane sedated) were imaged by IVIS spectrum for luminescence with an exposure time of 30 seconds.

The following animal work was approved by authorities for health and consumer protection in Hamburg, Germany (approval 41/17, 09/19). For Intracarotid injection, six-week-old female C57BL/6 j mice were anaesthetized, the left region of the neck was longitudinally opened, and the parotid gland was deviated laterally in order to expose the left carotid artery. The vagus nerve was carefully detached from the vessel, the carotid artery was temporarily ligated and a catheter (0.8 mm Ø) was retrogradely inserted and fixed with another ligature. HEK-293T CD63-NanoLuc EVs (1x10^11^) were slowly anterograde injected into the artery. Afterwards, the catheter was removed, the carotid artery was permanently ligated, and the skin was sutured. For intracardiac injection, six-weeks old female C57BL/6 j mice were anaesthetized and the left ventricle was punctured. The pulsatile entrance of bright-red oxygenated blood into the needle verified proper positioning and HEK-293T CD63-NanoLuc EVs (1x10^11^) were slowly injected. For Intracerebral injection, HEK-293T CD63-NanoLuc EVs (3x10^9^ in 2ul PBS) were stereotactically injected into the caudate/putamen. For comparison six-weeks-old female C57BL/6 j mice were injected with HEK-293T CD63-NanoLuc EVs (1x10^11^) IV Mice were sacrificed after 30 min, the different organs were harvested and stored in 2 ml Eppendorf tubes at −80°C until further use. Blood samples were immediately centrifuged at 500 x g for 7 min and 15,000 x g for 15 min at 4°C. Plasma samples were frozen at −80°C.

For NanoLuc measurement, tissues were lysed in 1 ml 0.1% TritonX-100 in PBS using a Qiagen Tissue Lyser II according to the manufacturer protocol. Tissue lysate was then diluted 1:10 in 0.1% TritonX-100 and 10 µl of tissue lysate was added into white-walled 96-well plates along with 30 μL Nano-Glo substrate diluted 1:50 in the provided buffer (Nano-Glo Luciferase Assay System: Promega), as per the manufacturer instructions. The luciferase intensity in each well was immediately measured using the GloMax® 96 Microplate Luminometer machine (Promega). For achieving sensitive quantification of EVs number in different tissues, values for known concentrations of EVs were used to make a standard curve and the concentration of EVs in different organs were calculated based on their respective RLU. When performing quantification or the relative quantification of the EVs, there are few technical aspects to take into account. For *in vivo* quantification, proper tissue lysis needs to be ensured. Another crucial factor is to test different dilutions of tissue homogenates until dilution factor correlates with luciferase activity. Using different equipment for measuring luminescence should furthermore be avoided as this can introduce variation. Lastly, substrates should be used according to the manufacturer recommendation.

## Results

### Screening luciferase species for efficient EV labelling

Bioluminescence assays can utilize a broad spectrum of luciferase enzymes. However, the enzymes used for EV labelling until now suffer from poor stability and low quantum yield, thus limiting their utility for various *in vitro* and *in vivo* applications. In addition, engineering strategies for EV loading used in previous studies often resulted in the labelling of only a minor fraction of the EV population [35]. Therefore, we concentrated on addressing these two critical issues.

**Figure 1.**
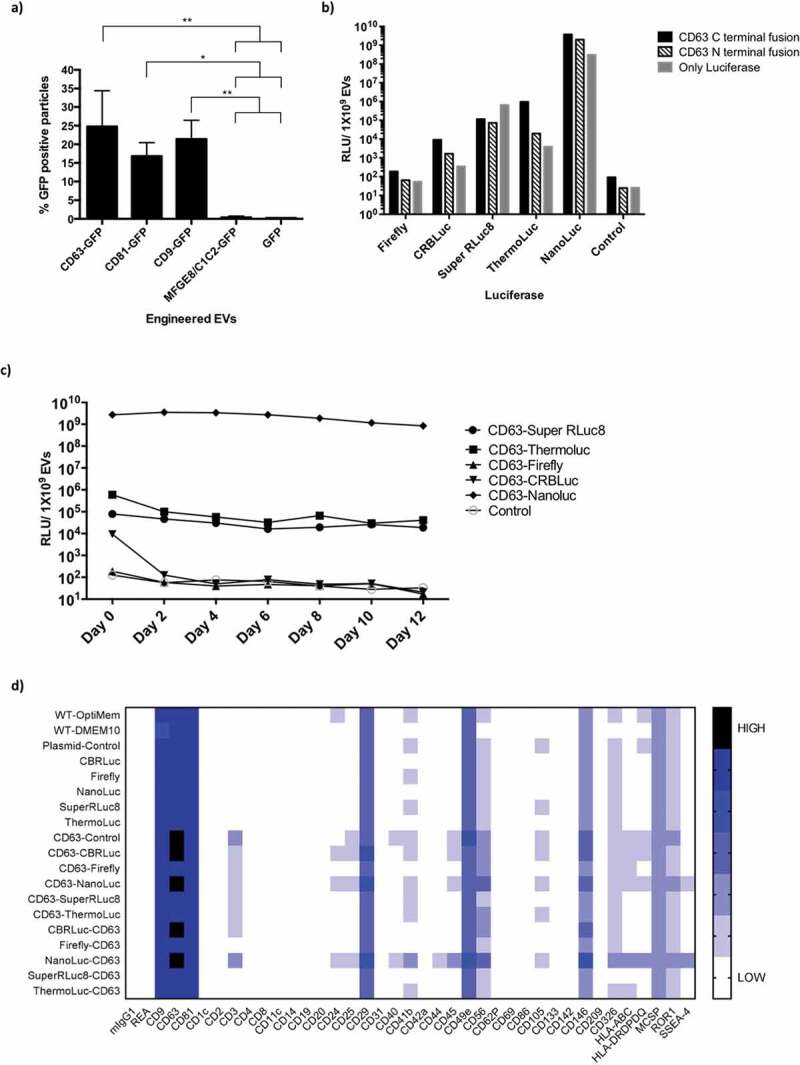
**Exploring strategies for bioluminescence labelling of EVs. a)** Percentage of GFP positive EVs, as determined by Fluorescence NTA of EVs purified from CM of HEK-293T cells transfected with pLEX-GFP-CD63, pLEX-GFP-CD81, pLEX-GFP-CD9, pLEX-GFP-MFGE8 or pLEX-GFP. Data was analysed by student’s test: *p < 0.05; **p < 0.01; ***p < 0.001. **b)** Relative luminescence activity, as determined by luciferase assays on 1×10^9^ engineered EVs purified from CM of HEK-293T cells transfected with pLEX-Firefly, pLEX-CD63-Firefly, pLEX-Firefly-CD63, pLEX-CRBLuc, pLEX-CD63-CRBLuc, pLEX-CRBLuc-CD63, pLEX-Super RLuc8, pLEX-CD63-Super RLuc8, pLEX-Super RLuc8-CD63, pLEX-ThermoLuc, pLEX-CD63-ThermoLuc, pLEX-ThermoLuc-CD63, pLEX-NanoLuc, pLEX-CD63-Nanoluc, pLEX-Nanoluc-CD63 or pLEX-CD63-EGFP (Control). **c)** Relative luminescence of 1×10^9^ luciferase-engineered EVs purified from CM of HEK-293T cells transfected with pLEX-CD63-Firefly, pLEX-CD63-CRBLuc, pLEX-CD63-Super RLuc8, pLEX-CD63-ThermoLuc, pLEX-CD63-Nanoluc or pLEX-CD63-EGFP, when incubated for different periods of time at 37°C in FBS. **d)** EVs engineered with different luciferases were phenotyped for 37 different surface markers by multiplex bead-based EV phenotyping assay.

To address the issue of loading efficiency as a limiting factor in EV labelling, we also investigated different EV sorting domains for their EV labelling efficiency. Upon transient transfection of HEK-293T cells, we found that CD9-, CD63- and CD81-GFP, all members of the tetraspanin family of membrane proteins and prominent EV markers, each labelled 15–25% of EVs with GFP, with CD63 being the most efficient labelling protein [35,36]. In contrast, overexpression of free GFP or MFGE8-GFP labelled less than 1% of HEK-293T EVs with GFP when analysed by NTA Figure 1a. These results correlated well together with our recent work on the systemic comparison of different EV sorting domains for EV engineering [33].

To identify potentially efficient luciferase species for EV labelling, we performed a literature search for enzymes that fulfilled the following two criteria [32,33,37,38]: First, stability in terms of half-life and pH sensitivity and, secondly, compatible signal wavelength and/or high intensity for better *in vivo* penetration. This identified five different luciferases of interest: NanoLuc, ThermoLuc, Firefly, Super Rluc8 and CBRLuc.

Based on the above analysis, HEK-293T cells were transfected with constructs expressing the identified luciferase species alone or fused to either the N- or C-terminus of CD63. Luciferase activity was assessed for 1×10^9^ EVs purified by ultra-filtration with subsequent size exclusion chromatography (SEC) Figure 1b. EVs engineered with NanoLuc fused to the N- or C-terminus of CD63 showed the highest relative luminescence units (RLU), followed by ThermoLuc CD63 C-terminal fusion. In contrast, Super RLuc8, Firefly and CRBLuc CD63 fusions all showed low activity Figure 1b. The expression of free luciferase proteins also resulted in EV loading, with NanoLuc or Super RLuc8 only showing the highest activity, suggesting a stochastic EV sorting ability of free protein in cells. Next, we measured the stability of the different EV loaded luciferase species over time in 50% foetal bovine serum (FBS) and PBS at 37°C. NanoLuc and Super RLuc8 engineered EVs showed enhanced luciferase stability over time as compared to other luciferase-engineered EVs and were stable in PBS and FBS for over 12 days and 10 days, respectively (Figure 1c and Supplementary Figure 1). In contrast, ThermoLuc engineered EVs showed stability only up to day 2 in both PBS and FBS. Importantly, engineering of EVs with the CD63 luciferase fusion proteins had no influence on their mode size (Supplementary Figure 2a) or measured EV surface protein composition (Figure 1d and Supplementary Figure 3) [34]. Importantly, the observed differences in surface expression of CD3, CD24, CD25, CD40 and CD105 were primarily due to high background associated with the assay, and slight differences were observed across different samples primarily due to limitations associated with the assay as previously shown [34]. In addition, HEK-293T cell viability was unaltered upon overexpression of CD63 and luciferase fusions (Supplementary Figure 4a and 4b).

**Figure 2.**
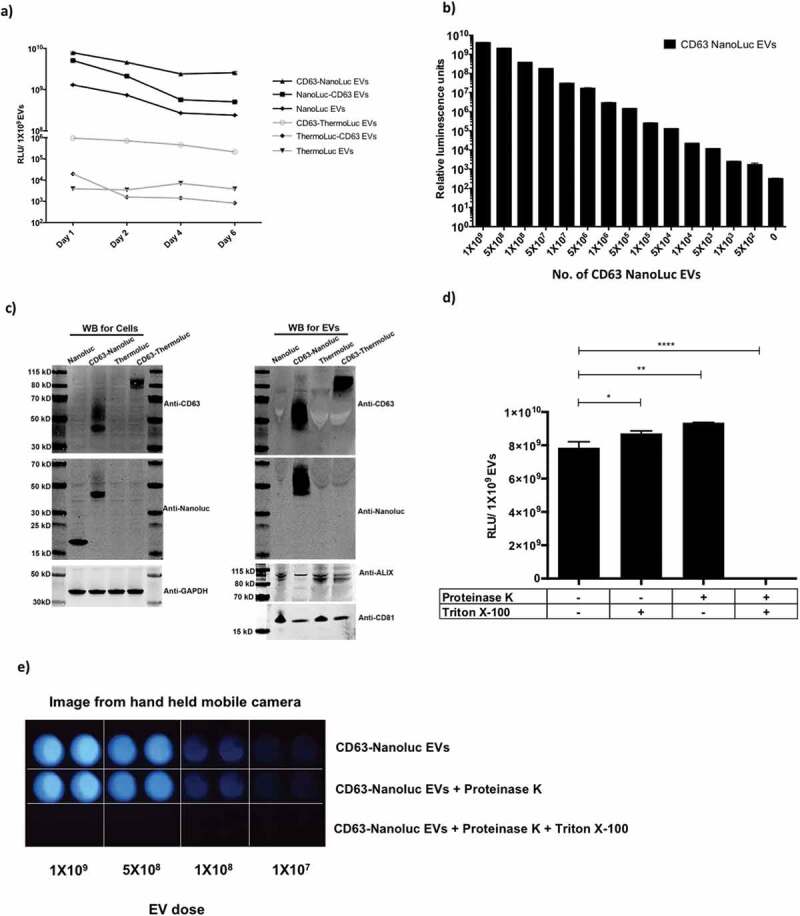
**Engineering EVs does not alter their characteristics. a)** Relative luminescence of 1×10^9^ luciferase-engineered EVs purified from CM of HEK-293T cells transfected with constructs pLEX-ThermoLuc, pLEX-CD63-ThermoLuc, pLEX-ThermoLuc-CD63, pLEX-NanoLuc, pLEX-CD63-Nanoluc or pLEX-Nanoluc-CD63, and incubated for different periods of time at 37°C in PBS. **b)** Relative luminescence of CD63-NanoLuc EVs at different concentrations. **c)** Western blots of cells and EVs purified from their CM following transfection with pLEX-NanoLuc (MW:19kDa), pLEX-CD63-Nanoluc (MW:44kDa), pLEX-ThermoLuc (MW:60kDa) or pLEX-CD63-ThermoLuc (MW:85kDa). Western blots were probed for NanoLuc, CD63, GAPDH (MW:36kDa), CD81 (MW:22kDa) and ALIX (MW:96kDa). **d)** Relative luminescence of 1×10^9^ CD63-NanoLuc engineered EVs following proteinase K 100µg/ml treatment with and without lysing the EVs in 0.1% TritonX-100 in PBS. Data was analysed by student’s test: *p < 0.05; **p < 0.01; ****p < 0.0001. e) Visualising EV bioluminescence by eye. Image taken by Handheld mobile camera of CD63 NanoLuc engineered EVs treated as indicated and exposed to substrate.

The initial screen with different luciferases suggested NanoLuc as the best candidate to efficiently label EVs due to its brightness and stability; however, ThermoLuc’s higher emission wavelength showed promising results upon applying an active EV loading strategy (Figure 2a and Supplementary Figure 4 c). Therefore, we concentrated on these two luciferase species for EV labelling to explore various applications. When comparing N- and C-terminal fusions of NanoLuc and ThermoLuc, enhanced stability and loading of both species were observed when fused to the C terminus of CD63 Figure 2a. In addition, NanoLuc engineered EVs showed a high dynamic range as the detection limit of CD63-NanoLuc engineered EVs is as low as 5000 EVs and exhibits a good correlation with dilution factor (R^2^) (Figure 2b and Supplementary Figure 5a). Similar linearity in luciferase activity was also observed with CD63-ThermoLuc EVs, with a detection limit of around 50,000 EVs (R^2^ = 0.9986) (Supplementary Figure 5b and 5c).

Western blot analysis further confirmed the expression of CD63-NanoLuc and CD63-ThermoLuc, along with other EV associated markers in both cells and EVs (Figure 2c). Interestingly, we observed enrichment of different sizes of CD63 fusion proteins in cells and EVs, which could suggest that certain glycosylated versions tend to sort into EVs more readily. Additionally, intact cup-shaped membrane vesicles of around 100 nm were observed in transmission electron microscopy of the EVs (Supplementary Figure 6a and 6b). To further validate the fact that the observed luminescence activity was from purified EVs engineered with luciferases, and not from protein aggregates, we performed Proteinase K treatments on purified EVs. We observed significant decay of luciferase activity only upon lysing CD63-NanoLuc EVs prior to Proteinase K treatment Figure 2d. Interestingly, the brightness of NanoLuc EVs after addition of substrate allowed us to visualise it by naked eye Figure 2e. Collectively, these results show that EVs can be efficiently loaded with luminal luciferase enzymes that are stable over time.

### Cells expressing CD63-NanoLuc secrete both vesicular and non-vesicular luciferase

**Figure 3.**
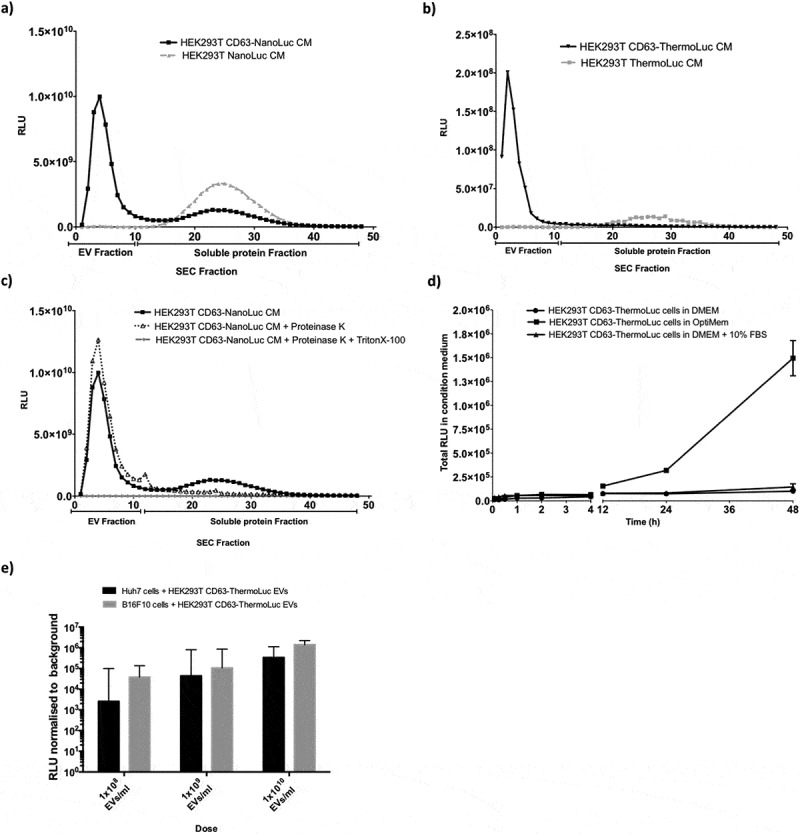
**CD63 fusion localizes luciferases to the EV lumen. a-b)** Luciferase activity in different UF-SEC fractions of the HEK-293T cell secretome following transfection with pLEX-CD63-NanoLuc or pLEX-NanoLuc (a), or pLEX-CD63-ThermoLuc or pLEX-ThermoLuc (b). **c)** NanoLuc activity in different UF-SEC fractions of the HEK-293T cell secretome following transfection with pLEX-CD63-NanoLuc following proteinase K treatment with and without lysing the EVs. **d)** Line graph depicting total RLU values observed at different time points in CM from HEK-293T cells stably expressing CD63-ThermoLuc in different culture conditions. **e)** Normalized RLUs from CD63-ThermoLuc labelled EVs in Huh7 or B16F10 recipient cells following treatment with different concentrations of EVs.

Previously, Hikita et al. [31] observed clear correlation between particle numbers and different tetraspanin NanoLuc fusions’ luminescence activity pre- and post-purification, which we fail to observe in our experiments (Supplementary Figure 6c). In light of this, we further investigated this discrepancy using SEC on the conditioned medium (CM) harvested from HEK-293T cells transfected with CD63-NanoLuc and -ThermoLuc fusions or the free luciferase protein-expressing constructs. As expected, the CM from CD63-NanoLuc transfected cells showed higher relative luciferase activity in the EV fraction, as compared that of free NanoLuc transfected cells. However, later SEC fractions containing soluble proteins showed luciferase activity in both free NanoLuc and CD63-NanoLuc CM Figure 3a. In contrast, CD63-ThermoLuc transfected cell CM showed activity almost exclusively in the EV enriched SEC fractions, while expression of free ThermoLuc led to the enrichment of luciferase protein only in the soluble protein fraction Figure 3b.

To further delineate the NanoLuc localisation in CM from cells transfected with CD63-NanoLuc and NanoLuc only, the different SEC fractions were treated with Proteinase K with and without lysing the EVs. The soluble protein fraction showed inhibition of luciferase activity upon Proteinase K treatment without lysis, whereas the EV fraction showed inhibition by Proteinase K only upon EV lysis (Figure 3c and Supplementary Figure 7a). We additionally observed a similar trend with CD9- and CD81-NanoLuc fusions, where a fraction of activity was observed in the soluble protein fraction and reduced by 50% upon proteinase K treatment (Supplementary Figure 7b and 7 c). Furthermore, upon comparing the normalised intensity of CD63-NanoLuc/CD63-ThermoLuc, and CD9-NanoLuc/CD9-ThermoLuc luciferase activity, ThermoLuc fusions showed less than 1% of the activity in the soluble protein fraction as compared to 50% for NanoLuc fusions (Supplementary Figure 7d, 7e and 7f). This highlights that upon overexpression of CD63-NanoLuc, CD81-NanoLuc and CD9-NanoLuc, NanoLuc exists in two distinct forms, one as an EV membrane-enclosed CD63 fusion and secondly as a free soluble protein.

This non-EV associated NanoLuc contributes to the luciferase activity detected in unpurified CM. This limits its use for certain applications specifically involving release kinetics of EVs from cells *in vitro* and *in vivo*. Since signal in the EV fractions was unaffected by Proteinase K treatment alone, purified NanoLuc EVs can be applied for various *in vitro* and *in vivo* applications, while ThermoLuc labelling can be used in both unpurified and purified settings. Importantly, we also observed an increase in RLU per particle with ThermoLuc–tetraspanin fusion after purification. We speculate that this is mainly due to the presence of non-EV-associated protein aggregates, which are therefore removed upon purification. All studies utilising luciferase species to track EVs thus need to investigate the possibility of free luciferase in the CM very carefully.

### *Bioluminescent labelling of EVs offers diverse* in vitro *applications*

To explore the potential applications of luciferase-engineered EVs in various *in vitro* assays, we utilised CD63-ThermoLuc EV labelling to analyse the release kinetics of EVs from producer cells in a high-throughput and cost-effective manner. Here, as an example of this application, we tested the effect of different cell culture conditions on the EV release and uptake kinetics patterns of EVs over 48 hours. To avoid inter-experimental variation due to the transfection of luciferase-tetraspanin fusion proteins, cells carrying stably integrated ThermoLuc- or NanoLuc-tetraspanin fusion genes were used for all further experiments. Both DMEM and DMEM + 10% FBS showed similar EV release profiles after 24 hours; however, after 24 hours cells in OptiMEM showed substantially increased EV secretion and cells grown in DMEM alone displayed the lowest EV secretion Figure 3d. Moreover, we observed similar EV release profiles upon tracking EV release with CD81- and CD9-ThermoLuc engineered cells, thus confirming that the observed effect was not CD63 dependant (Supplementary figure 8a-b). Furthermore, we confirmed these observations with NTA by measuring particles per ml at different time points and observed an almost 100% correlation between NTA values and luciferase activity (Supplementary Figure 9a-9d). Importantly culturing cells in DMEM only showed signs of reduced cell viability, in contrast to those in OptiMEM and DMEM+10% FBS (data not shown). These differences could be because of the activation of certain signalling pathways involved in EVs release upon culturing cells in OptiMEM.

Apart from EV release kinetics, we explored the application of luciferase-engineered EVs for measuring the quantitative uptake of EVs in recipient cells. CD63-ThermoLuc EVs were purified and added in a range of doses to different recipient cells. The cell lysate was analysed 2 hours post-treatment in order to measure EV uptake Figure 3e. Our quantifications showed dose-dependent EV uptake in recipient cells for both the Huh7 hepatocellular carcinoma cell line and the B16F10 mouse melanoma cell line. Moreover, CD63-NanoLuc EVs showed similar EV uptake pattern in Huh7 cells (Supplementary figure 9e).

### *Cord blood MSC EVs show fast* in vivo *distribution kinetics and rapid plasma clearance*

Given the high dynamic range of detection with CD63-NanoLuc engineered EVs, these are particularly well suited for determining the *in vivo* pharmacokinetics and distribution profile of exogenous EVs. In light of the therapeutic potential of EVs, a clinically relevant cell source was selected to understand the *in vivo* dynamics of EVs. Therefore, mice were injected intravenously with 1×10^11^ CD63-NanoLuc cord blood MSC-derived EVs and euthanized at different time points ranging from 5 min to 24 hours post injection. Organs and plasma were subsequently harvested after perfusion, lysed and analysed for luciferase activity. To quantify the number of administered EVs in different organs, we determined the RLU per particle of CD63-NanoLuc cord blood MSC EVs and used that value to derive the total number of EVs in different tissues from background-normalised values (Supplementary Figure 10a). Importantly, we did not observe any major fluctuations in NanoLuc activity upon spiking in CD63-NanoLuc EVs into different tissue lysates as compared to adding the EVs into PBS (Supplementary Figure 10b). The detection limit of the assay was determined by measuring luminescence of different organs harvested from mice injected with PBS.

**Figure 4.**
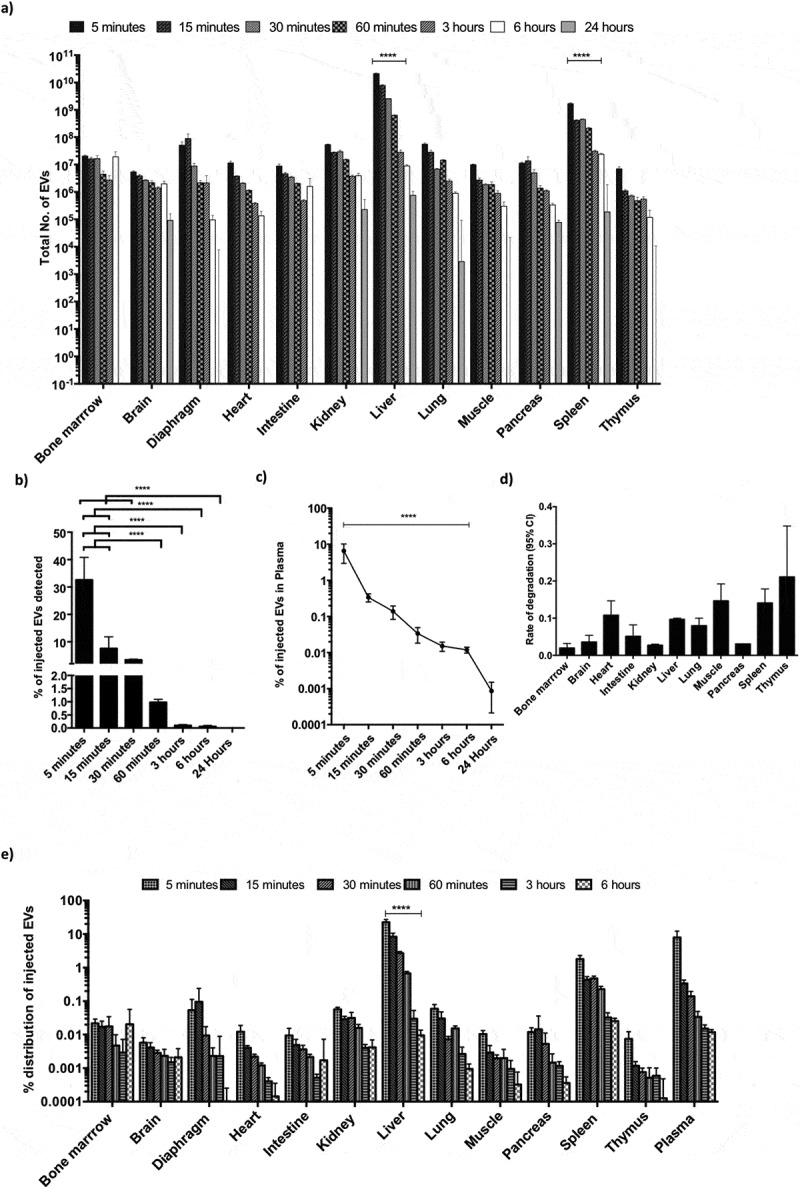
**Dynamic biodistribution of CD63-NanoLuc engineered EVs derived from cord blood MSCs stably expressing CD63-NanoLuc. a)** Total number of EVs detected in different organs at various time points following IV administration of 1×10^11^ EVs (n = 4). **b)** Cumulative percentage of injected EVs detected in assayed organs after IV administration of 1×10^11^ EVs (n = 4). **c)** Line graph displaying the percentage of injected EVs detected in plasma at different time points after IV administration of 1×10^11^ EVs (n = 4). d) Calculated 95% CI of rate of signal degradation in different organs over time. **e)** Percentage of injected EVs detected in different organs over time after IV administration of 1×10^11^ EVs (n = 4). Data was analysed by two-tailed student’s test: *p < 0.05; **p < 0.01; ***p < 0.001; ****p < 0.0001.

Strikingly, EVs accumulated in all the organs analysed, with levels peaking at 5 min post injection and declining over time (Figure 4a, 4b, 4c and Supplementary Figure 11a-b). EVs were primarily taken up by the liver and spleen, which significantly contributed to 9% and 1% of the injected EVs, and 90% and 10% of the detected EVs at 5 min post injection, respectively (Figure 4e and Supplementary Figure 11b). We could account for 30% of the injected EVs in the organs analysed at 5 min post injection, with levels gradually decreasing at later time points. This suggested rapid *in vivo* uptake of CD63 positive EVs by most internal organs, followed most likely by intracellular degradation Figure 4(a-d).

In addition, the EV levels detected at different time points in the various tissues were inversely correlated with EV levels in plasma. Here, 90% of the injected dose had been cleared after 5 min and was down to 0.1% 30 min post injection, suggesting an EV plasma half-life of 1.2–1.3 mins (95% confidence interval (CI)) upon applying one phase decay equation Figure 4c. This further highlights the rapid clearance of the EVs from plasma due to their uptake into tissues. Importantly, no differences were observed in NanoLuc EV biodistribution with or without perfusion at 5 min and 6 hours post injection (Supplementary Figure 12), corroborating the interpretation of our results as rapid plasma clearance.

### In vivo *biodistribution of EVs is influenced by route of administration.*

**Figure 5.**
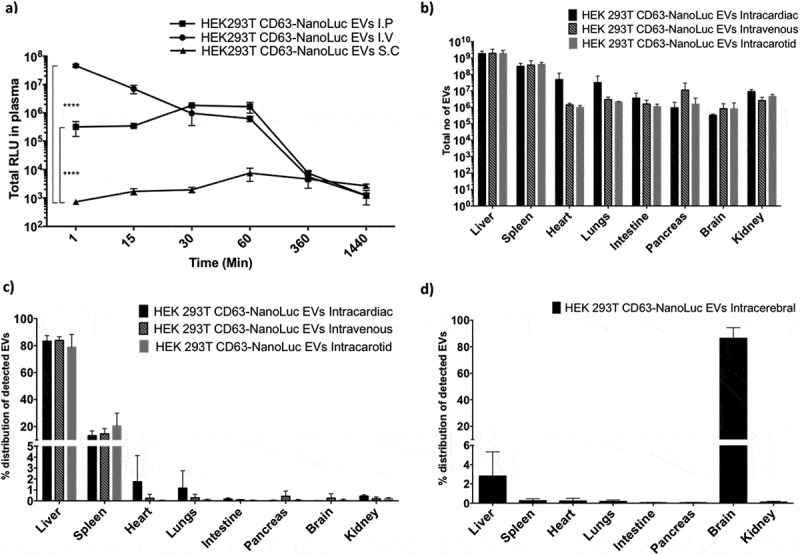
**Route of administration influences EV biodistribution**. *in vivo* biodistribution and serum pharmacokinetics of EVs purified from HEK-293T cells stably expressing CD63-NanoLuc transgene. **a)** Line graph displaying total NanoLuc EVs levels detected in plasma at different time points after IV, IP or SC administration of 1×10^11^ EVs (n = 3). Data was analysed by two-tailed student’s test: ****p < 0.0001. **b-c)** Total number of EVs detected (b) and percentage distribution (c) of detected EVs in different organs 30 min after intravenous, intracarotid or intracardiac administration of 1×10^11^ EVs (n = 4). Data was analysed by two-tailed student’s test: ****p < 0.0001. **d)** Percentage distribution of detected EVs in different organs 30 min post intracerebral administration of 3×10^9^ EVs (n = 5).

In order to further understand the plasma pharmacokinetics of EVs, different routes of injection were analysed. For further experiments, HEK-293T cells were utilized as a model cell line as they are widely used in the EV engineering field. For this, 1×10^11^ CD63-NanoLuc engineered HEK-293T EVs were administered to animals intravenously (IV), intraperitoneally (IP) or subcutaneously (SC) and blood was sampled periodically over 48 hours Figure 5a. Based on the luciferase activity detected, IV and IP displayed similar plasma pharmacokinetics, whereas SC administration showed only modest levels of luciferase activity in circulation. Both IV and IP administered EVs showed similar accumulation in the liver (Supplementary Figure 13a).

We further analysed intracarotid, intracardiac, peroral (P.O.) and intracerebral delivery of EVs to investigate if these would change EV distribution patterns. However, neither arterial injections altered the biodistribution or plasma half-life of the EVs (Figure 5b, 5c and Supplementary Figure 13b). These results suggest that EVs’ association with certain blood components determines there *in vivo* biodistribution and that the first capillary bed that EVs encounter does not influence the distribution.

Interestingly, peroral (P.O.) delivery of 2×10^11^ MSC CD63-NanoLuc EVs restricted the distribution of EVs to the stomach, with no signs of systemic uptake of the EVs as levels were below the detection limit (Supplementary Figure 13c).

Similarly, intracerebral injection of 3×10^9^ HEK-293T CD63-NanoLuc EVs limited EV biodistribution primarily to the brain (Figure 5d and Supplementary Figure 13d). Interestingly, we observed 2–3% of EVs being taken up by the liver, we speculate that this could be due to a small population of EVs crossing the BBB to peripheral circulation and enriching in the liver. Importantly, the difference in the dose for intracerebral administration was primarily due to restrictions in injection volume.

### ThermoLuc EV labelling enables non-invasive in vivo tracking of EVs in real time

**Figure 6.**
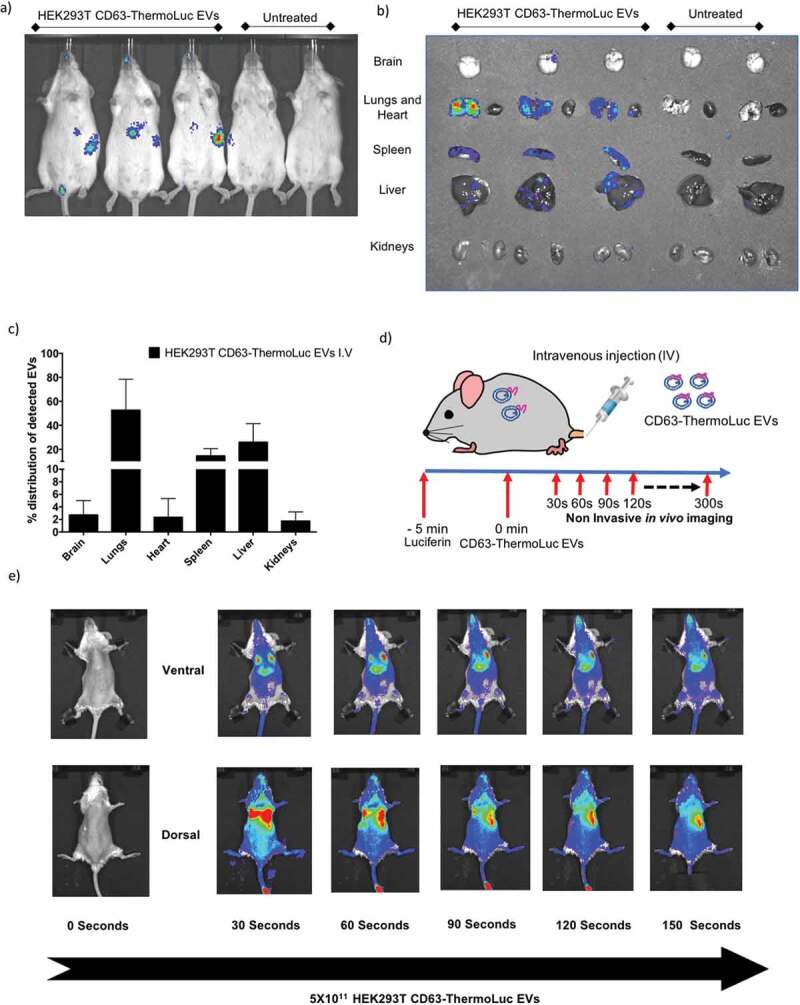
**Bioluminescence labelling for live *in vivo* tracking of EVs. a-b)** Non-invasive live animal imaging (a) and ex vivo imaging of organs (b) in animals IV injected either with 5×10^11^ HEK-293T cell-derived CD63-ThermoLuc EVs or PBS 30 mins post injection (n = 4), all animals were injected with D-Luciferin (I.P) prior to imaging. **c)** Percentage distribution of detected CD63-ThermoLuc EVs, as determined by ex vivo quantification. **d)** Method for visualising EV biodistribution in real time. **e)** Non-invasive imaging of mice seconds after IV administration of CD63-ThermoLuc EVs (n = 2).

Although NanoLuc labelling of EVs allowed for high sensitivity determination and quantification of the *in vivo* distribution of EVs, poor substrate distribution *in vivo*, toxicity and shorter emission wavelength impose a challenge for its application in non-invasive *in vivo* imaging [40]. For these reasons, ThermoLuc labelled EVs have an advantage for non-invasive *in vivo* imaging, as these display a high emission wavelength and no reported substrate toxicity. Animals intravenously administered with 5×10^11^ CD63-ThermoLuc labelled HEK-293T EVs showed a clear distribution profile to liver and spleen at 30 min post-injection by non-invasive live imaging Figure 6a. However, the intensity of the signal from the liver, spleen and lungs made it difficult to determine EV biodistribution in other internal organs by non-invasive imaging. Therefore, we performed *ex vivo* imaging of other organs to determine the complete biodistribution pattern of CD63-ThermoLuc EVs (Figure 6b, 6c and Supplementary Figure 13e). This analysis showed greater ThermoLuc EV accumulation in the lungs as compared to NanoLuc EVs. This could be due to an increased aggregation of EVs, as higher doses of ThermoLuc EVs were injected into animals in order to allow for the detection of the signal [27].

As EVs showed a short circulation half-life and a rapid body-wide uptake profile within 5 min post injection, we set out to determine the distribution pattern of EVs just seconds after administration. To trace EVs *in vivo* in real time, we administered luciferin 5 min prior to injecting of 5×10^11^ CD63-ThermoLuc EVs to allow for body-wide distribution of the substrate. Upon imaging animals periodically every 30 seconds after the injection, a rapid distribution of EVs to lungs was observed within 30 seconds of injection. This was followed by redistribution primarily to the liver after 60 seconds post injection, and to spleen after 90 seconds, as well as an increasing diffuse signal from the whole animal Figure 6e. In agreement with our earlier results, this clearly demonstrated a rapid body-wide distribution profile upon systemic bolus injection of EVs, where lungs, liver and spleen were the primary target organs.

### *EV subpopulations differ in their* in vivo *biodistribution.*

**Figure 7.**
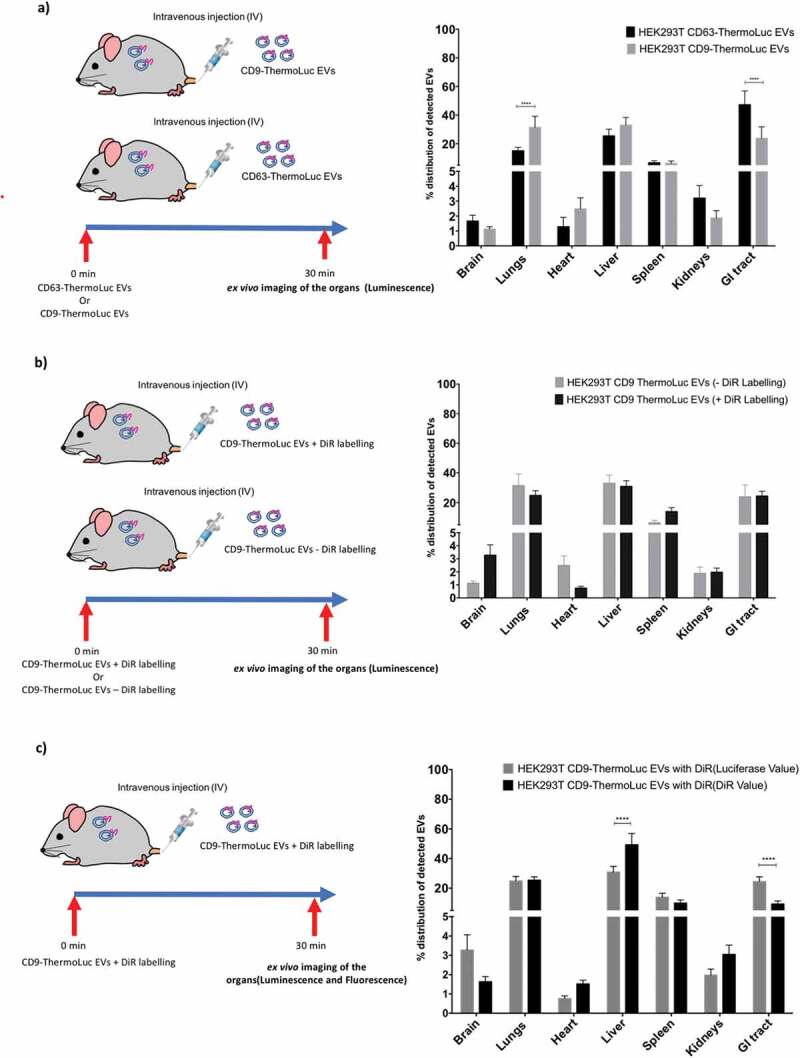
**EVs subpopulations differ in their *in vivo* biodistributions. a)** Percentage distribution of EVs in different organs from animals injected with CD63-ThermoLuc or CD9-ThermoLuc EVs 30 mins post IV administration (n = 3). Data was analysed by two tailed student’s test: ****p < 0.0001. **b)** Percentage distribution of detected EVs in different organs from animals injected with CD9-ThermoLuc EVs with DiR or without DiR labelling (n = 3). **c)** Percentage distribution of EVs in different organs from animals injected with CD9-ThermoLuc EVs labelled with DiR, as determined by both DiR Fluorescence and ThermoLuc luminescence (n = 3). Data was analysed by two-tailed student’s test: ****p < 0.0001. Values presented here were normalized to animals injected with PBS only and all animals were injected with D-Luciferin (I.P) prior to imaging.

Proteomic analysis and EV characterization studies have demonstrated that the tetraspanins CD9, CD63 and CD81 are the most abundant proteins on EVs. However, we and others have recently shown that the different tetraspanins exist on a different subpopulation of EVs [35,41]. To investigate the role of different EV subpopulations, as distinguished by their tetraspanin expression profile, with respect to their *in vivo* biodistribution, we IV injected mice with 2.5×10^11^ EVs purified from HEK-293T expressing CD63- or CD9-ThermoLuc and imaged the animals at 30 mins post injection. Both tetraspanin-engineered EV populations showed a similar biodistribution trend towards the liver and spleen. However, the relative distribution of CD63 positive EVs was significantly enhanced in the GI tract and reduced in the lungs. In addition, CD63 positive EVs showed a tendency towards enrichment in the brain and kidneys as compared to CD9 labelled EVs (Figure 7a and Supplementary Figure 14). These differences in biodistribution illustrate how different EV populations can potentially differ in their *in vivo* uptake.

### *Lipophilic dye labelling affects* in vivo *biodistribution of EVs*

Lipophilic dye-based labelling is a widely used method for tracking EVs *in vitro* and *in vivo* [27]. To assess if lipophilic dye-based labelling affects EV biodistribution *in vivo*, we injected mice with either 2.5x10^11^ unlabelled CD9-ThermoLuc HEK-293T EVs or labelled with near infrared lipophilic dye (DiR). We then assessed only organ luminescence signals *ex vivo* by IVIS to determine the relative distribution of ThermoLuc EVs 30 min after injection. Although distribution to liver and lungs was largely unaltered, the relative distribution of the EVs to brain and spleen was increased upon DiR labelling Figure 7b.

In addition, we compared DiR labelling and ThermoLuc labelling for determining the relative distribution of the EVs. For this, we injected animals with CD9-ThermoLuc EVs labelled with DiR. Upon assessing *ex vivo* ThermoLuc luminescence and DiR fluorescence in the different organs harvested from animals at 30 mins post injection, we observed lack of correlation in certain organs between the ThermoLuc luminescence signal and the fluorescence signal from DiR. In contrast to luciferase activity, DiR displayed greater distribution to the liver and reduced relative distribution to the GI tract and brain (Figure 7c and Supplementary Figure 15). These results suggest that DiR labelling of EVs may affect their *in vivo* biodistribution, most likely by altering the EV surface and by the transfer of dye to cell membranes.

## Discussion

In this study, we have explored a bioluminescence platform for efficient endogenous labelling of EVs. By engineering producer cells to express various tetraspanin-luciferase fusion proteins, we have shown the utility of this approach for various *in vitro* and *in vivo* applications. In addition, we have utilised this method to label different subpopulations of EVs and identified variations in their *in vivo* biodistribution. This work represents the first report of EV luciferase labelling that can achieve sensitive quantification of the number of EVs distributed *in vivo*.

In the past decade, bioluminescence imaging has emerged as a versatile tool with diverse applications in both basic biology and drug delivery research *in vitro* and *in vivo*. Because of its great sensitivity, others have tested endogenous bioluminescence labelling of EVs by genetically modifying their source cells. The primary issue with these previous studies has been the luciferase proteins utilised and the strategy used for sorting luciferase proteins into EVs. Most of these studies have simply overexpressed free luciferase or made use of MFGE8 (Lactadherin) fusions in producer cells [13,19,20,22,29], both of which we found to be less efficient than tetraspanin-fusion-based sorting. In addition, a system for sensitive cargo-based labelling of EVs would allow to specifically track different subpopulations of vesicles as compared to passively loaded luciferase enzymes into EVs. Furthermore, these reports have utilised Gaussia or Renilla for EV detection, and while these luciferases are stable, they lack high signal intensity. For instance, Super Rluc8, a Renilla analogue with enhanced activity, showed only modest activity when compared to the ThermoLuc and NanoLuc CD63 fusion proteins used in this study. In addition, we also tested AkaLuc for EV labelling, which was recently described for imaging single cells *in vivo* [42]. Unfortunately we failed to detect the EVs *in vivo* using our experimental setup.

NanoLuc is a recently discovered luciferase enzyme that is both small and bright. Previously, Hikita et al. and Kojima et al. have both utilised this for EV labelling and high-throughput quantification of EV release [12,31]. However, Hikita et al. showed higher NanoLuc signal values in the conditioned medium than in purified EVs and the study lacked substantial reasoning for this. We observed a similar phenomenon and have demonstrated experimentally that this is most likely due to the presence of non-vesicular NanoLuc protein, the origin of which could be due to its intracellular cleavage or due to crushed vesicles both of which would result in the presence of free protein. Therefore, the use of NanoLuc luciferase for EV research requires further optimisation before being applied to the evaluation of EV release *in vitro* and *in vivo*. As it stands, NanoLuc imposes a high risk of tracing the activity of free protein instead of EV associated protein.

With this knowledge, we utilised our platform to address certain key unanswered questions in EV research. First, we showed the complete *in vivo* pharmacokinetic profile of the EVs, which demonstrated their rapid systemic uptake in all internal organs and muscle tissues. In addition, we also observed EVs being cleared after their accumulation in different tissues, although the rate of degradation was variable in different organs. This is most likely because different cell types take up and process the EVs differently in different organs. Importantly, when EVs were injected directly into the vein or artery, the specific route of injection had no influence on EV biodistribution. For example, following intracarotid injections, brain enrichment was observed to be similar to that following IV injections. This indicates that the levels of EVs in CNS are determined by factors other than the initial tissue-specific fenestrations that EVs encounter. Furthermore, delivery routes without direct access to the blood network failed to show any systemic bioavailability of EVs. For example, when delivered orally, EVs were primarily distributed to the stomach or stuck with the food and no signal was detected in different organs despite the limit of detection of the assay being as low as 5000 EVs.

Interestingly, the distribution profile of EVs determined by NanoLuc and ThermoLuc correlated remarkably well with that determined by radionuclide-labelled EVs [25]. This suggests that NanoLuc and ThermoLuc hold great potential to expand access to the reliable determination of *in vivo* biodistribution of EVs. Importantly, NanoLuc allows for high sensitivity quantification of EV distribution *in vivo* in a relatively high-throughput manner. However, although NanoLuc offers a broad dynamic range, *in vivo* quantification can only be performed *ex vivo*, as poor substrate distribution, emission wavelength and toxicity impose challenges to its application for non-invasive imaging.

Therefore, for non-invasive tracking of EVs, we used ThermoLuc labelling due to its high signal intensity and higher emission wavelength, which penetrates tissue more readily [32]. By utilising this method, we could demonstrate the *in vivo* distribution of EVs in real time directly after injection for the first time.

Advances in the purification and quantification of EVs have resulted in the description of various distinct subtypes of EV populations on a physiochemical level. However, their behaviour *in vivo* has remained unexplored due to challenges of labelling specific EVs populations [43,44]. Cargo-based labelling has an advantage, as it allows for population-specific labelling of EVs. Here, we have labelled three different tetraspanin expressing EV populations and demonstrated differences in their EV secretion patterns and showed *in vivo* biodistributions profile of two different tetraspanin populations. These results may help to explain the phenomenon behind the differences observed in biodistribution of EVs derived from different cell sources [27]. Utilizing this approach has great potential, as overexpression or engineering population-specific marker loci in the genome with fusions to the above-mentioned luciferases could be used to label different populations of EVs *in vivo* [31].

We have further shown that exogenous labelling strategies, based on lipophilic fluorescent dyes, over- or underestimates relative distribution in different organs. In addition, they can alter EV biodistribution to certain organs from that observed using only the luciferase labelling system in the current experimental setting. This may be because dyes alter EV surface properties via their association with proteins or lipids exposed on the EV surface. Importantly, exogenous labelling strategies do not reflect EV half-life, as these agents are highly stable and resistant to degradation, but do offer the flexibility of staining EVs without the need of modifying the producer cell, especially in the cases of human plasma or tissue-derived EVs [28,44,45]. In addition, as free contrast or dye can produce false-positive results, a purification step after labelling is crucial and adds variability between experiments.

Overall, NanoLuc and ThermoLuc labelling of EVs holds great potential for various *in vivo* and *in vitro* applications. In addition, it can enable the simultaneous detection of different subpopulations of EVs *in vivo*, which may aid in our understanding of different sub-populations and their behaviour *in vivo*. Apart from monitoring therapeutic EVs, with one simple modification, this platform offers great potential for tracking tumour-derived EVs both *in vivo* and *in vitro* and thus could aid in the development of anti-tumour therapies.

## Supplementary Material

Supplemental Material
