# Supplementary material for: Quantification of extracellular vesicles in vitro and in vivo using sensitive bioluminescence imaging
Source: J Extracell Vesicles. 2020 Aug 21;9(1):1800222. doi: 10.1080/20013078.2020.1800222 (PMC7481830; doi:10.1080/20013078.2020.1800222)
Supplement: Supplemental Material [file ZJEV_A_1800222_SM6146.zip › Supplementary/Supplementary/figure legends.docx]

**Supplementary Figure 1. a-b)** Relative luminescence of 1x10^9^ luciferase engineered EVs purified from HEK293T cells transfected with constructs expressing CD63 N-terminus luciferase fusions, when incubated for different periods of time at 37˚C in a) PBS or b) FBS. **c-d)** Relative luminescence of EVs purified from HEK293T cells transfected with constructs expressing free luciferase, when incubated for different periods of time at 37˚C in c) PBS or d) FBS. **e)** Relative luminescence of EVs purified from HEK293T cells transfected with constructs expressing CD63 C-terminus luciferase fusions, when incubated for different amounts of time at 37˚C in e) PBS. **f)** Biological half-life at 37˚C in FBS of the luciferase enzyme upon passive or active loading into EVs.

**Supplementary Figure 2. a)** Mode EV size, as determined by NTA of EVs purified from HEK293T cells transfected with pLEX-ThermoLuc, pLEX-CD63-ThermoLuc, pLEX-NanoLuc or pLEX-CD63-Nanoluc.

**Supplementary Figure 3.** EV surface protein signature determination by multiplex bead-based assays of EVs purified from HEK293T cells transfected with pLEX-Firefly, pLEX-CD63-Firefly, pLEX-Firefly-CD63, pLEX-CRBLuc, pLEX-CD63-CRBLuc, pLEX-CRBLuc-CD63, pLEX-Super RLuc8, pLEX-CD63-Super RLuc8, pLEX-Super RLuc8-CD63, pLEX-ThermoLuc, pLEX-CD63-ThermoLuc, pLEX-ThermoLuc-CD63, pLEX-NanoLuc, pLEX-CD63-Nanoluc, pLEX-Nanoluc-CD63 or pLEX-CD63-EGFP. Values depict the relative levels of different surface markers on EVs normalised to control IgG staining.

**Supplementary Figure 4. a)** Fold increase in CD63-APC MFI over untreated cells, as determined by flow cytometry on HEK293T cells transfected with pLEX-Firefly, pLEX-CD63-Firefly, pLEX-Firefly-CD63, pLEX-CRBLuc, pLEX-CD63-CRBLuc, pLEX-CRBLuc-CD63, pLEX-Super RLuc8, pLEX-CD63-Super RLuc8, pLEX-Super RLuc8-CD63, pLEX-ThermoLuc, pLEX-CD63-ThermoLuc, pLEX-ThermoLuc-CD63, pLEX-NanoLuc, pLEX-CD63-Nanoluc, pLEX-Nanoluc-CD63 or pLEX-CD63-EGFP 48 hours post transfection. **b)** Cell viability, as determined by DAPI staining of the cells transfected with pLEX-Firefly, pLEX-CD63-Firefly, pLEX-Firefly-CD63, pLEX-CRBLuc, pLEX-CD63-CRBLuc, pLEX-CRBLuc-CD63, pLEX-Super RLuc8, pLEX-CD63-Super RLuc8, pLEX-Super RLuc8-CD63, pLEX-ThermoLuc, pLEX-CD63-ThermoLuc, pLEX-ThermoLuc-CD63, pLEX-NanoLuc, pLEX-CD63-Nanoluc, pLEX-Nanoluc-CD63 or pLEX-CD63-EGFP, and analysed by flowcytometry 48 hours post transfection. **c)** Emission spectrum profile of HEK293T CD63-ThermoLuc and CD63-NanoLuc EVs. Shaded regions represent the peak intensity of each luciferase.

**Supplementary Figure 5.** NanoLuc and ThermoLuc Engineered EVs offer broad dynamic range. **a)** Linear regression analysis of CD63-NanoLuc EVs number versus RLU. **b)** Relative luminescence activity of CD63-ThermoLuc EVs. **c)** Linear regression analysis of CD63-ThermoLuc EVs number versus RLU.

**Supplementary Figure 6.** Transmission electron microscopy of EVs purified from HEK293T cells transfected with **a)** pLEX-NanoLuc and **b)** pLEX-CD63-Nanoluc confirmed the cup shaped morphology of the EVs. **c)** Percentage difference in RLU of 1x10^8^ particles in CM of HEK293T cells transfected with pLEX-NanoLuc, pLEX-CD9-NanoLuc, pLEX-CD63-NanoLuc, pLEX-CD81-NanoLuc, pLEX-ThermoLuc, pLEX-CD9-ThermoLuc, and pLEX-CD63-ThermoLuc, before and after purification with Size exclusion chromatography. Data was analysed by two tailed student’s test: *****p* < 0.0001.

**Supplementary Figure 7 a)** NanoLuc activity in different fractions (300µl) of the secretome derived from HEK293T cells transfected with pLEX-NanoLuc and the effect of Proteinase K on NanoLuc activity with and without lysis with TritonX100. **b, c)** NanoLuc activity in different fractions (1ml) of secretome derived from HEK293T cells transfected with b) pLEX-CD9-NanoLuc or c) pLEX-CD81-NanoLuc and effect of Proteinase K on NanoLuc activity with and without lysis. **d)** ThermoLuc activity in different fractions (1ml) of secretome derived from HEK293T cells transfected with pLEX-CD63-ThermoLuc or pLEX-CD9 ThermoLuc. **e and f)** Normalised luciferase activity of secretome derived from HEK293T cells transfected with e) pLEX- CD63 NanoLuc and pLEX-CD63 ThermoLuc, f) pLEX-CD9 NanoLuc and pLEX-CD9 ThermoLuc.

**Supplementary Figure 8.** **a-b)** Line graphs depicting the total detected RLUs at different time points in CM from HEK293T cells stably expressing CD9- (a) and CD81-ThermoLuc (b) in different culture conditions.

**Supplementary Figure 9 a)** Graph depicting particle per ml determined by NTA in condition medium over time from HEK293T cells stably expressing CD63-, CD9- or CD81-ThermoLuc cultured in OptiMEM. Normalised luciferase activity and normalised particle per/ml in condition medium over time from HEK293T cells stably expressing **b)** CD63- or **c)** CD81- or **d)** CD9-ThermoLuc cultured in OptiMEM. **e)** Determination of *in vitro* cellular uptake of NanoLuc labelled HEK293T EVs in recipient Huh7 cells. Graph depicting total RLU observed in the cells normalised to background.

**Supplementary Figure 10. a)** Relative luminescence activity of cord blood MSC CD63-NanoLuc EVs at indicated concentrations. **b)** Effect of tissue lysates on luciferase activity of NanoLuc EVs. 2X10^6^ HEK293T CD63-NanoLuc EVs were spiked in 100mg of tissue lysate from various organs before measurement.

**Supplementary Figure 11. a-b)** Number of EVs/mg tissue detected (a) and percentage distribution of detected EVs (b) in various organs at different time points after IV administration of 1x10^11^ EVs derived from cord blood MSCs stably expressing CD63-NanoLuc (n=4).

**Supplementary Figure 12. a-b)** Effect of perfusion prior to euthanising the animals on the number of EVs detected in different organs at 5 minutes (a) and 6 hours (b) after IV administration of 1x10^11^ EVs derived from cord blood MSCs stably expressing CD63-NanoLuc (n=4).

**Supplementary Figure 13.**  **a)** Luminescence signal detected in Liver and Brain after 30 minutes post Intravenous, Intraperitoneal or sub cutaneous administration of 1X10^11^ EVs derived from HEK stably expressing CD63 NanoLuc (n=4). **b)** Percentage of injected EVs detected in plasma after 30 minutes post Intravenous, Intracarotid or Intracardiac administration of 1X10^11^ EVs derived from HEK stably expressing CD63 NanoLuc. (n=4). **c)** RLU/mg of tissue detected in different organs at 30 minutes after per oral administration of 2X10^11^ EVs derived from MSC stably expressing CD63-NanoLuc (n=4). **d)** Total No. of EVs detected in different organs at 30 minutes after intracerebral administration of 1X10^11^ EVs derived from HEK293T stably expressing CD63 NanoLuc (n=5). **e)** Photon per second detected in different organs at 30 minutes after intravenous administration of 5X10^11^ EVs derived from HEK293T stably expressing CD63 ThermoLuc (n=4), all values presented are normalised to background.

**Supplementary Figure 14. a)** Ex vivo Luminescence imaging of organs from animals IV injected with 2.5x10^11^ HEK293T CD63-ThermoLuc EVs, at 30 minutes post injection. **b)** Ex vivo Luminescence imaging of organs from animals IV injected with 2.5x10^11^ HEK293T CD9-ThermoLuc EVs, at 30 minutes post injection.

**Supplementary Figure 15. a)** Ex vivo fluorescence imaging of organs from animals IV injected with 2.5x10^11^ HEK293T CD9-ThermoLuc EVs labelled with DiR, at 30 minutes post injection. **b)** Ex vivo fluorescence imaging of organs from animals IV injected with 2.5x10^11^ HEK293T CD9-ThermoLuc EVs labelled with DiR, at 30 minutes post injection.
