# Supplementary figures and images for: Quantification of extracellular vesicles in vitro and in vivo using sensitive bioluminescence imaging
Source: J Extracell Vesicles. 2020 Aug 21;9(1):1800222. doi: 10.1080/20013078.2020.1800222 (PMC7481830; doi:10.1080/20013078.2020.1800222)

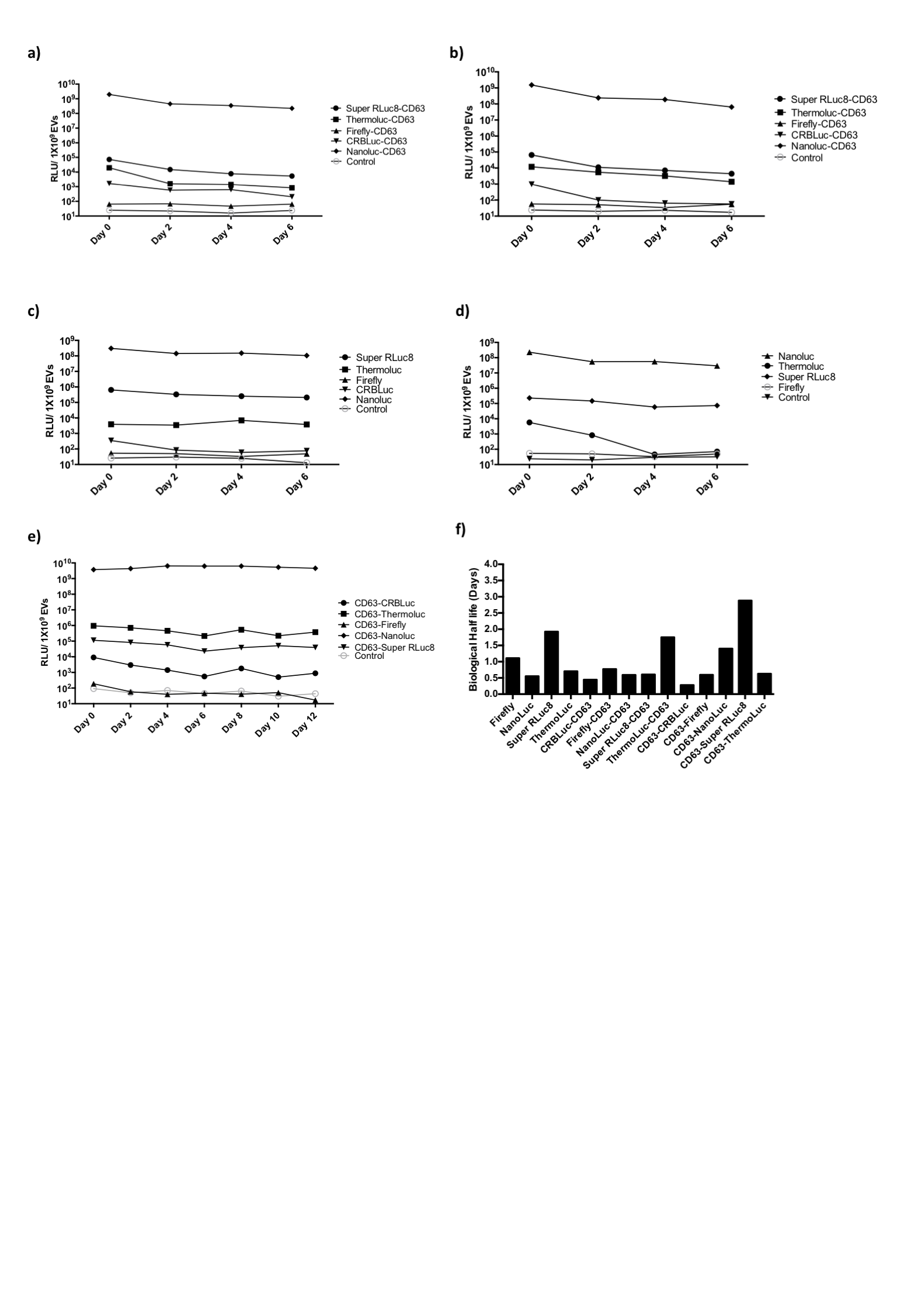

Supplement: Supplemental Material [file ZJEV_A_1800222_SM6146.zip › Supplementary/Supplementary/Supplementary Figure 1.tiff]

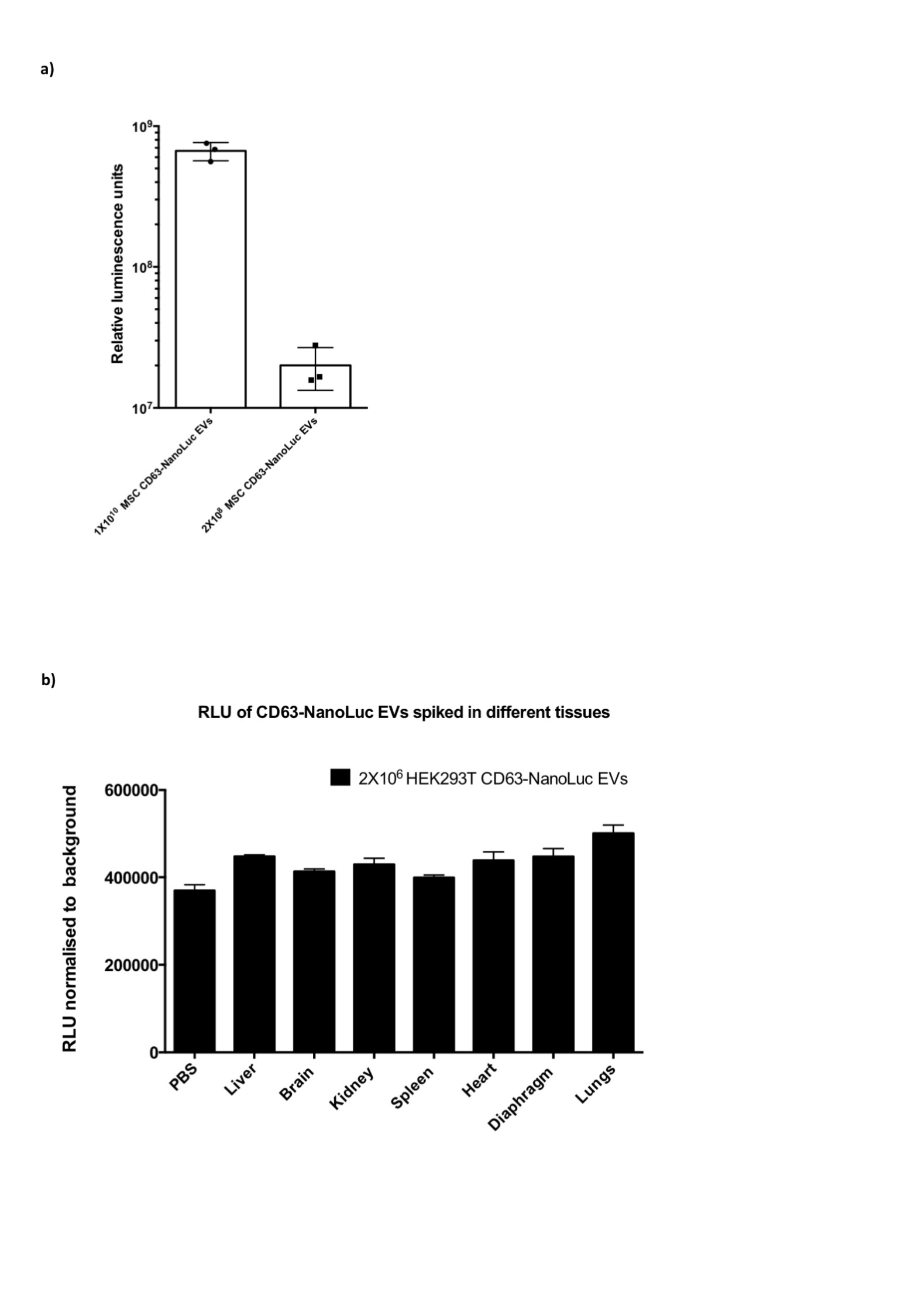

Supplement: Supplemental Material [file ZJEV_A_1800222_SM6146.zip › Supplementary/Supplementary/Supplementary Figure 10.tiff]

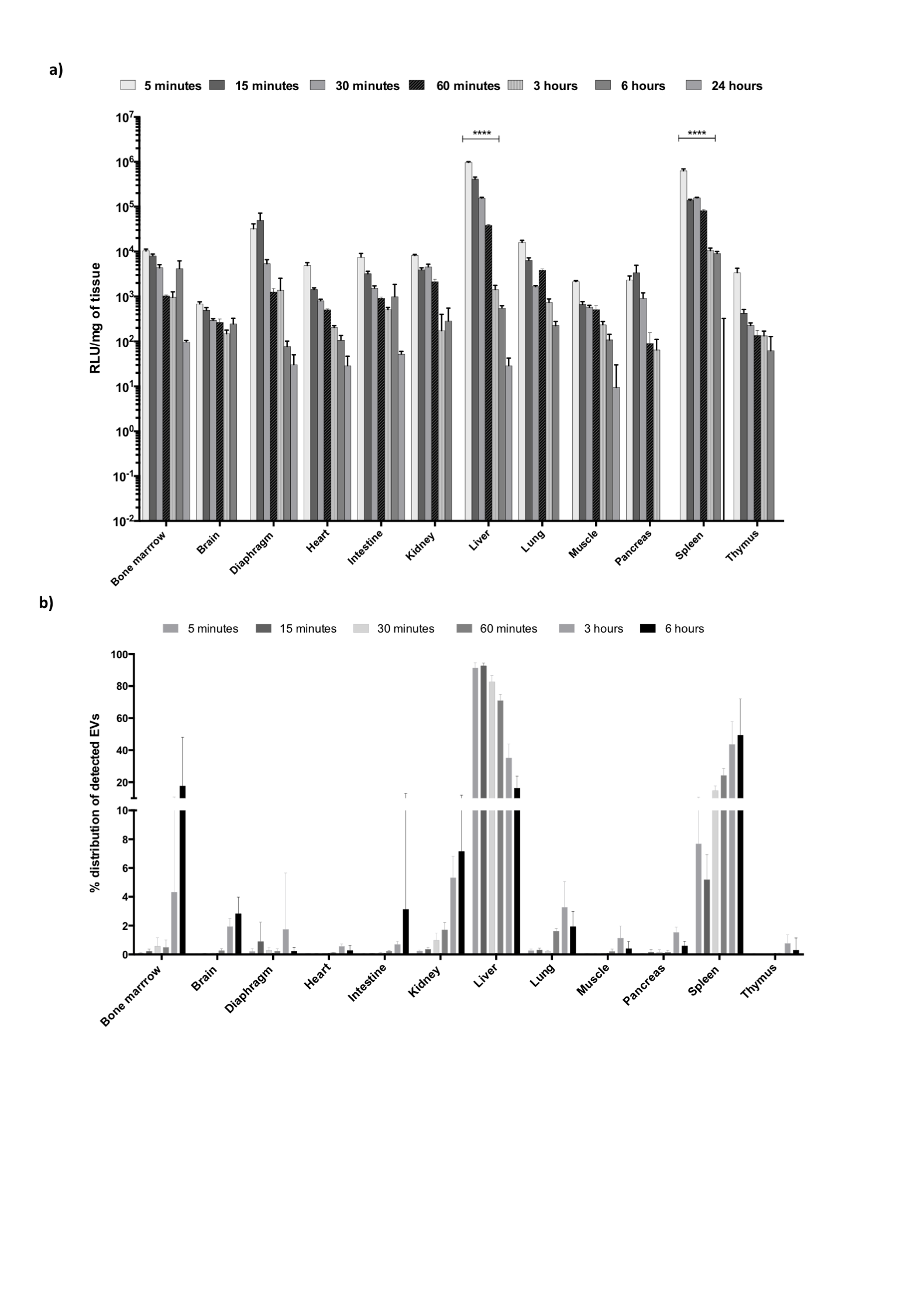

Supplement: Supplemental Material [file ZJEV_A_1800222_SM6146.zip › Supplementary/Supplementary/Supplementary Figure 11.tiff]

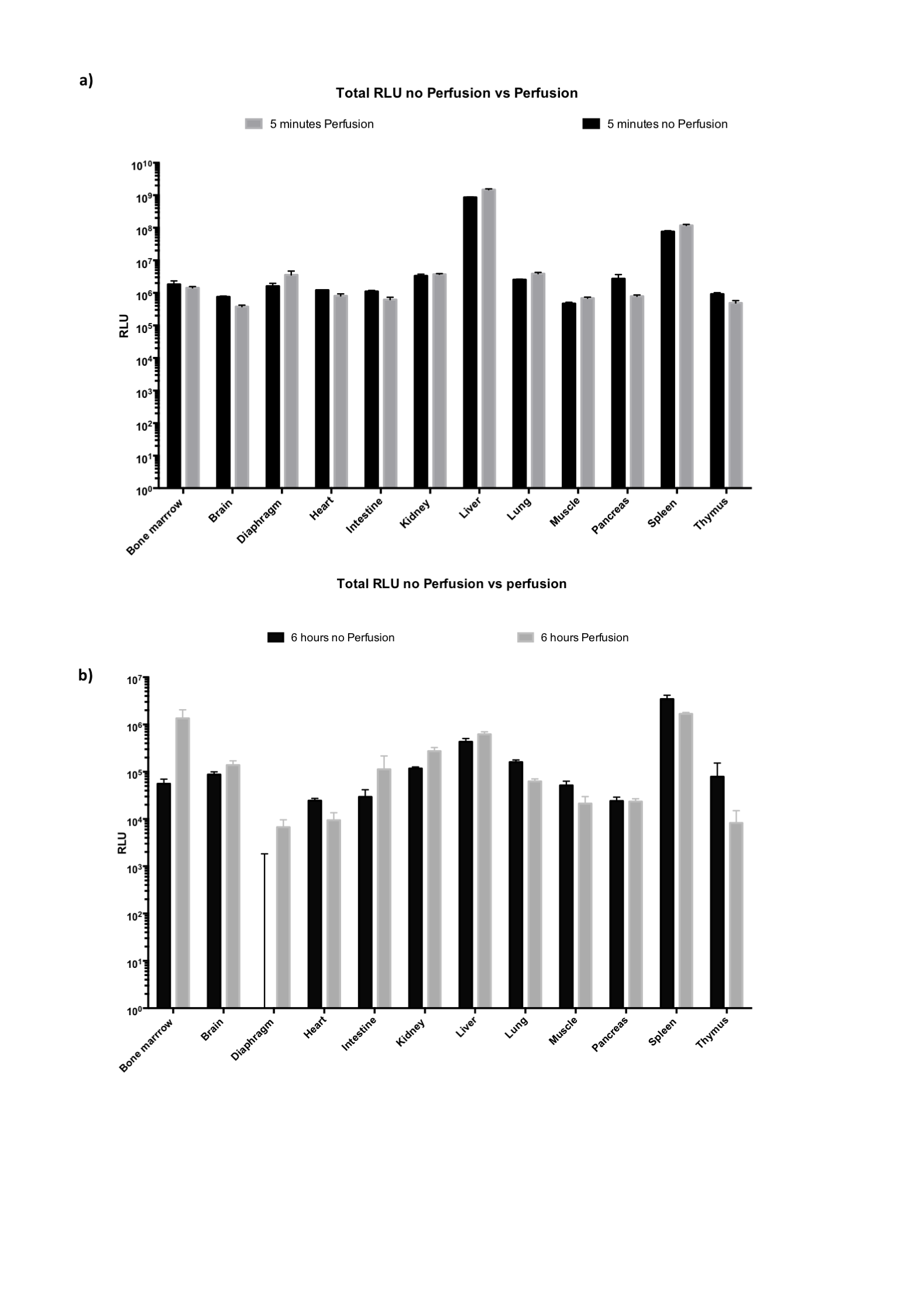

Supplement: Supplemental Material [file ZJEV_A_1800222_SM6146.zip › Supplementary/Supplementary/Supplementary Figure 12.tiff]

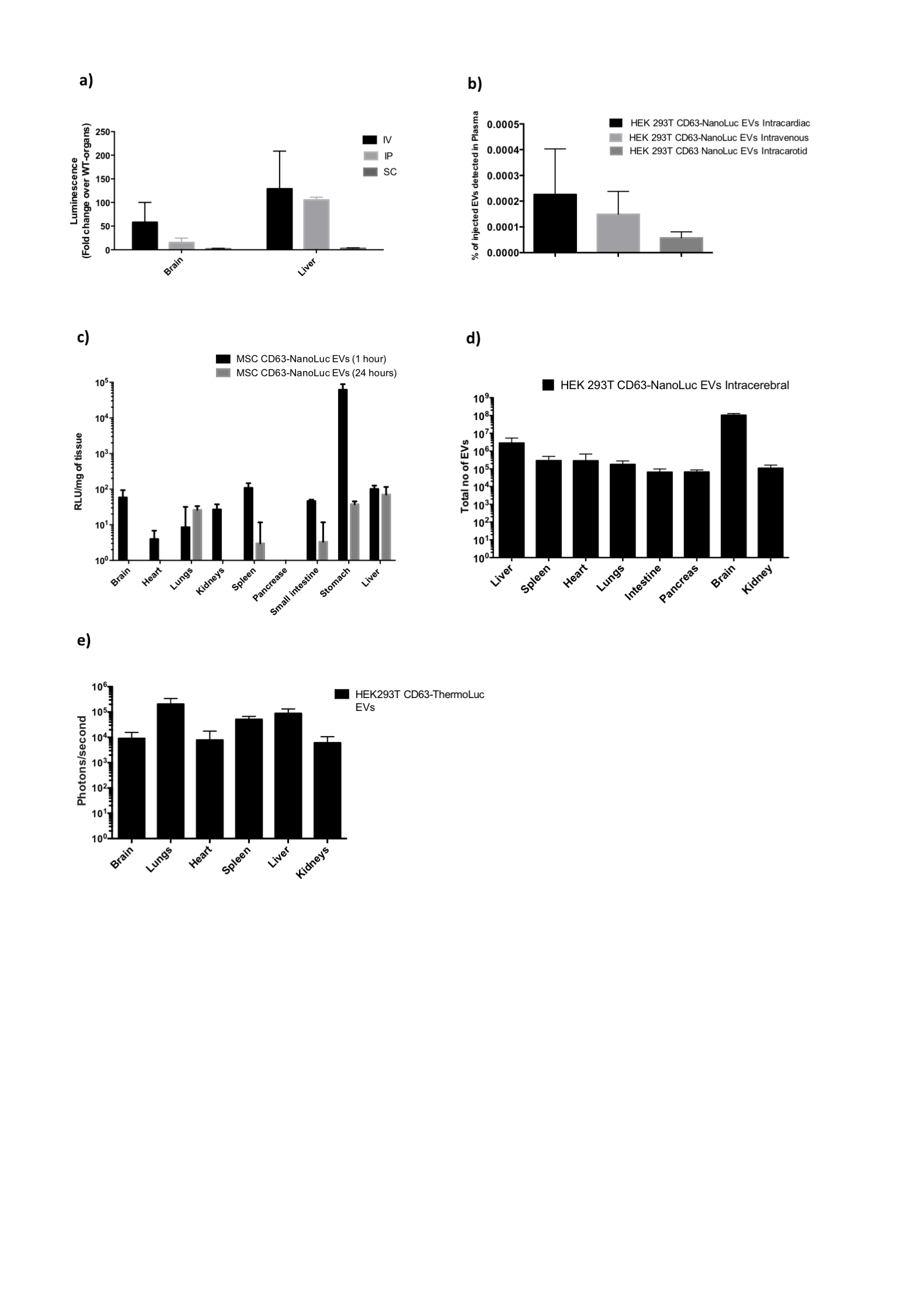

Supplement: Supplemental Material [file ZJEV_A_1800222_SM6146.zip › Supplementary/Supplementary/Supplementary Figure 13.tiff]

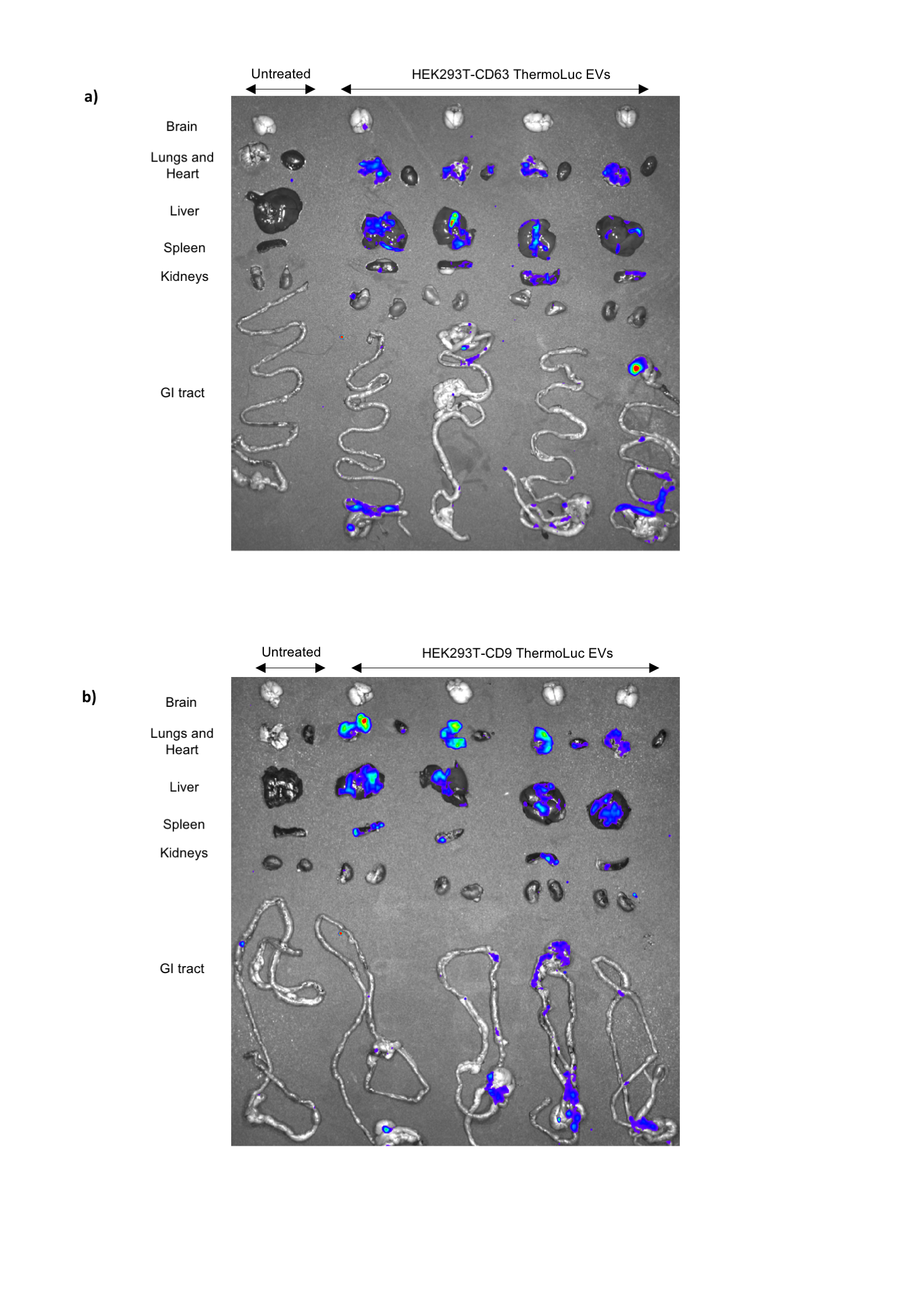

Supplement: Supplemental Material [file ZJEV_A_1800222_SM6146.zip › Supplementary/Supplementary/Supplementary Figure 14.tiff]

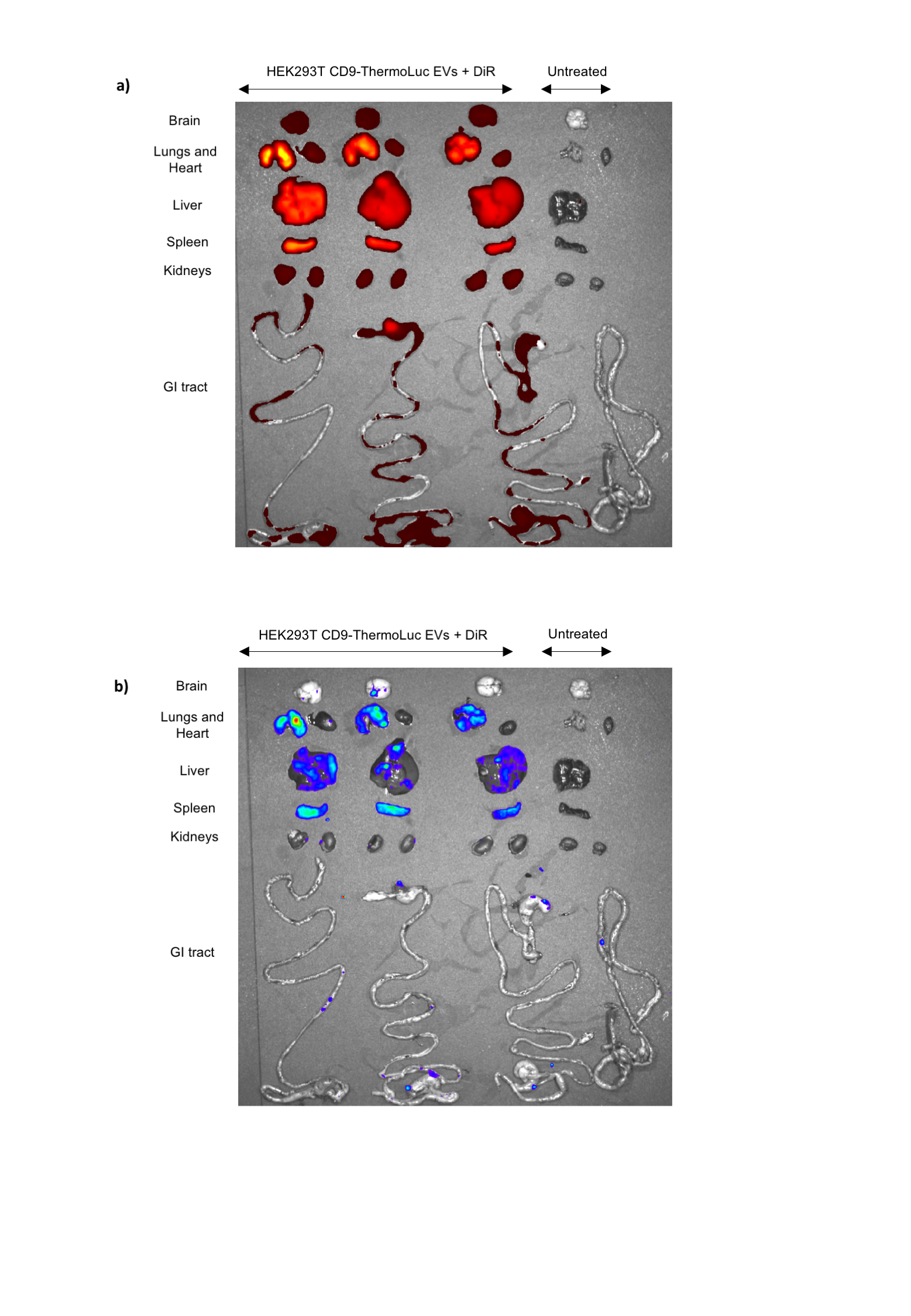

Supplement: Supplemental Material [file ZJEV_A_1800222_SM6146.zip › Supplementary/Supplementary/Supplementary Figure 15.tiff]

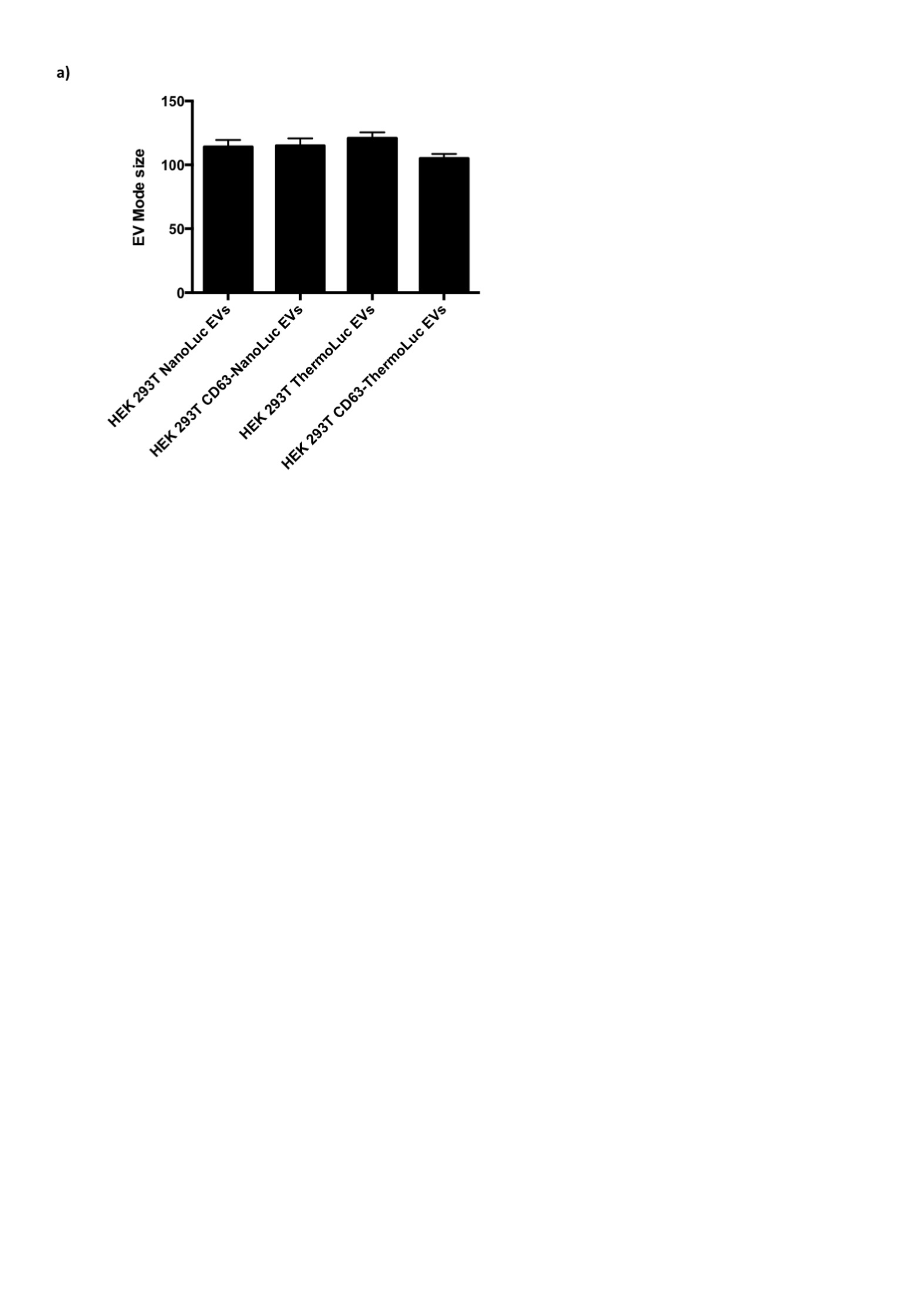

Supplement: Supplemental Material [file ZJEV_A_1800222_SM6146.zip › Supplementary/Supplementary/Supplementary Figure 2.tiff]

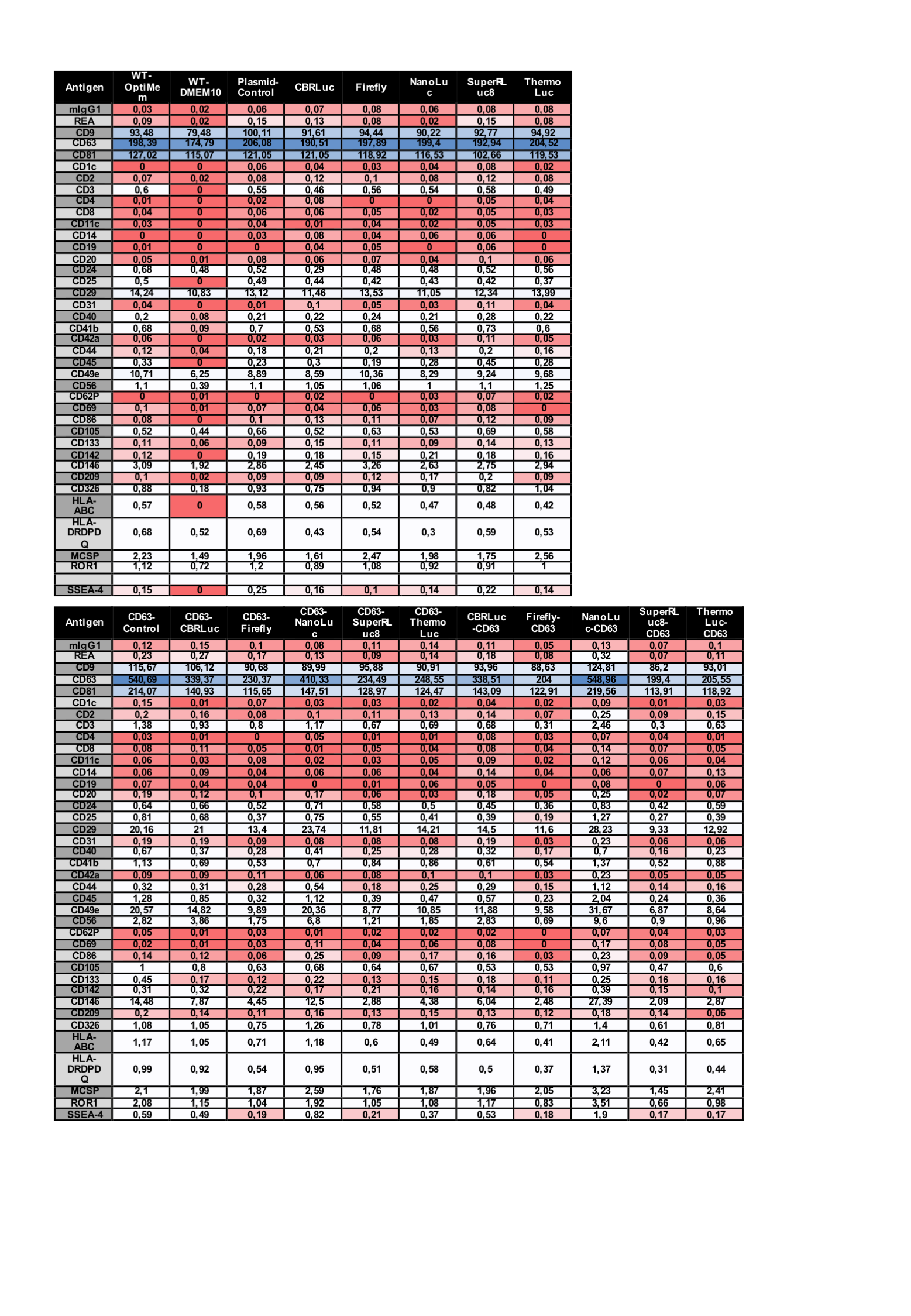

Supplement: Supplemental Material [file ZJEV_A_1800222_SM6146.zip › Supplementary/Supplementary/Supplementary Figure 3.tiff]

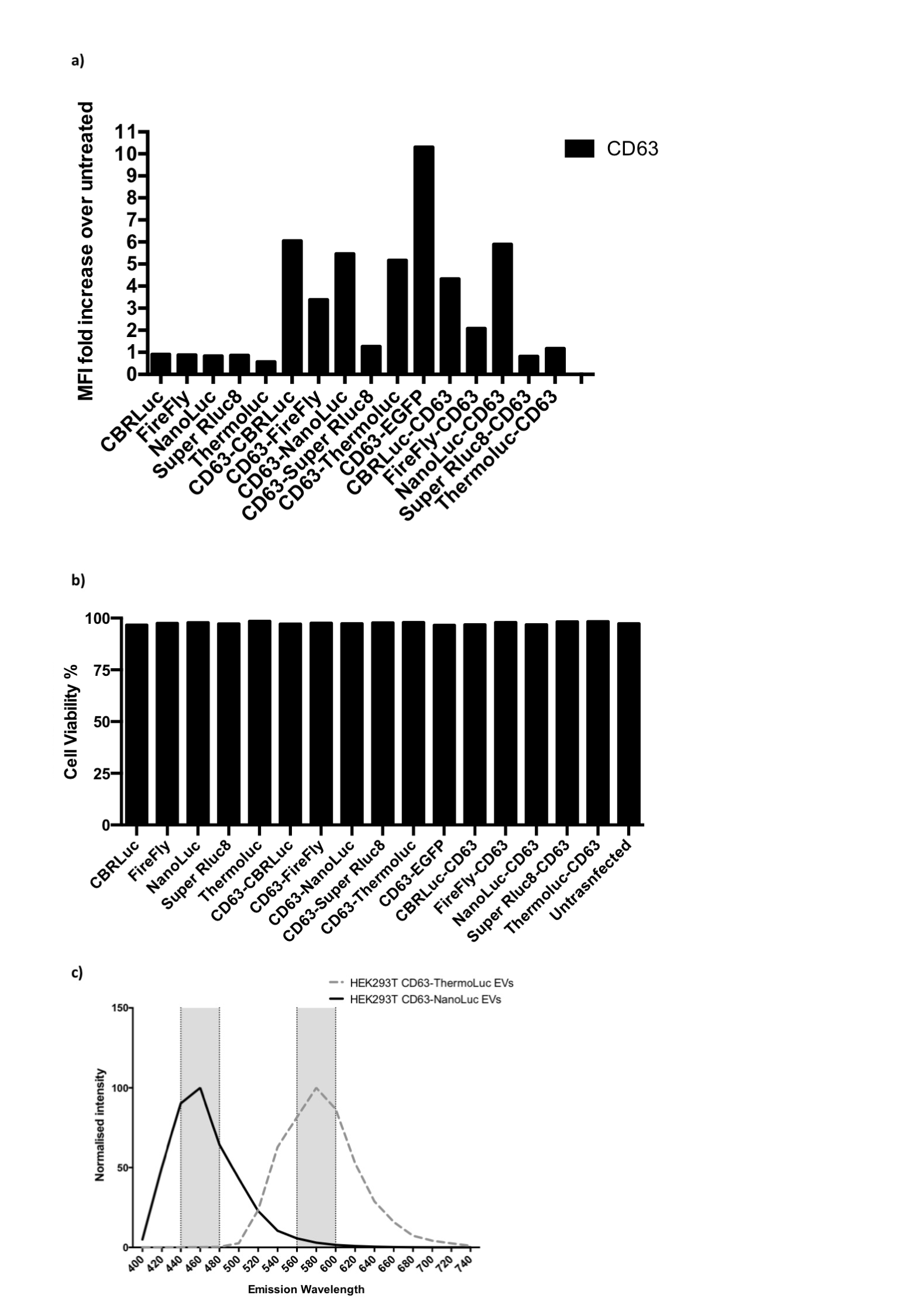

Supplement: Supplemental Material [file ZJEV_A_1800222_SM6146.zip › Supplementary/Supplementary/Supplementary Figure 4.tiff]

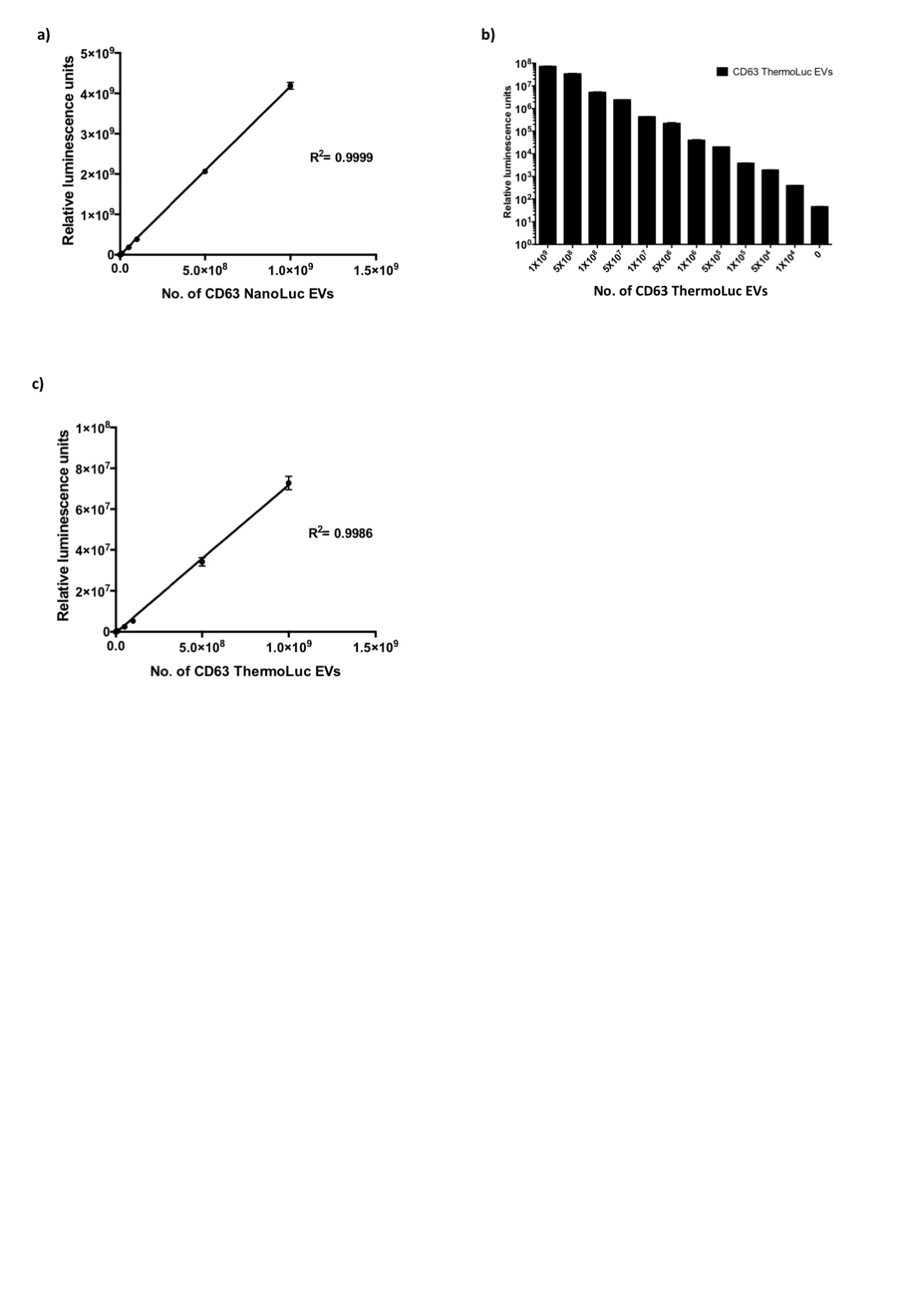

Supplement: Supplemental Material [file ZJEV_A_1800222_SM6146.zip › Supplementary/Supplementary/Supplementary Figure 5.tiff]

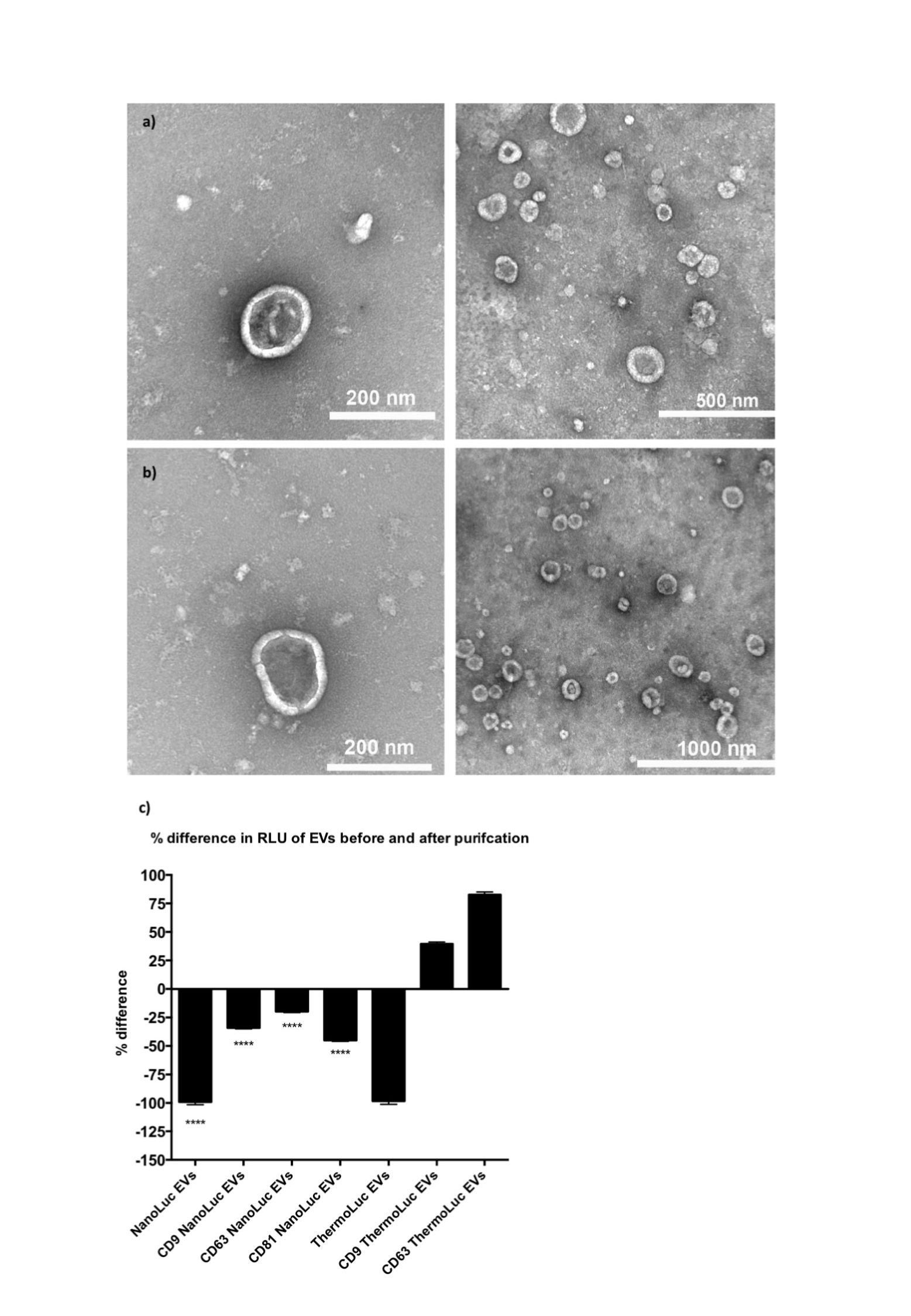

Supplement: Supplemental Material [file ZJEV_A_1800222_SM6146.zip › Supplementary/Supplementary/Supplementary Figure 6.tiff]

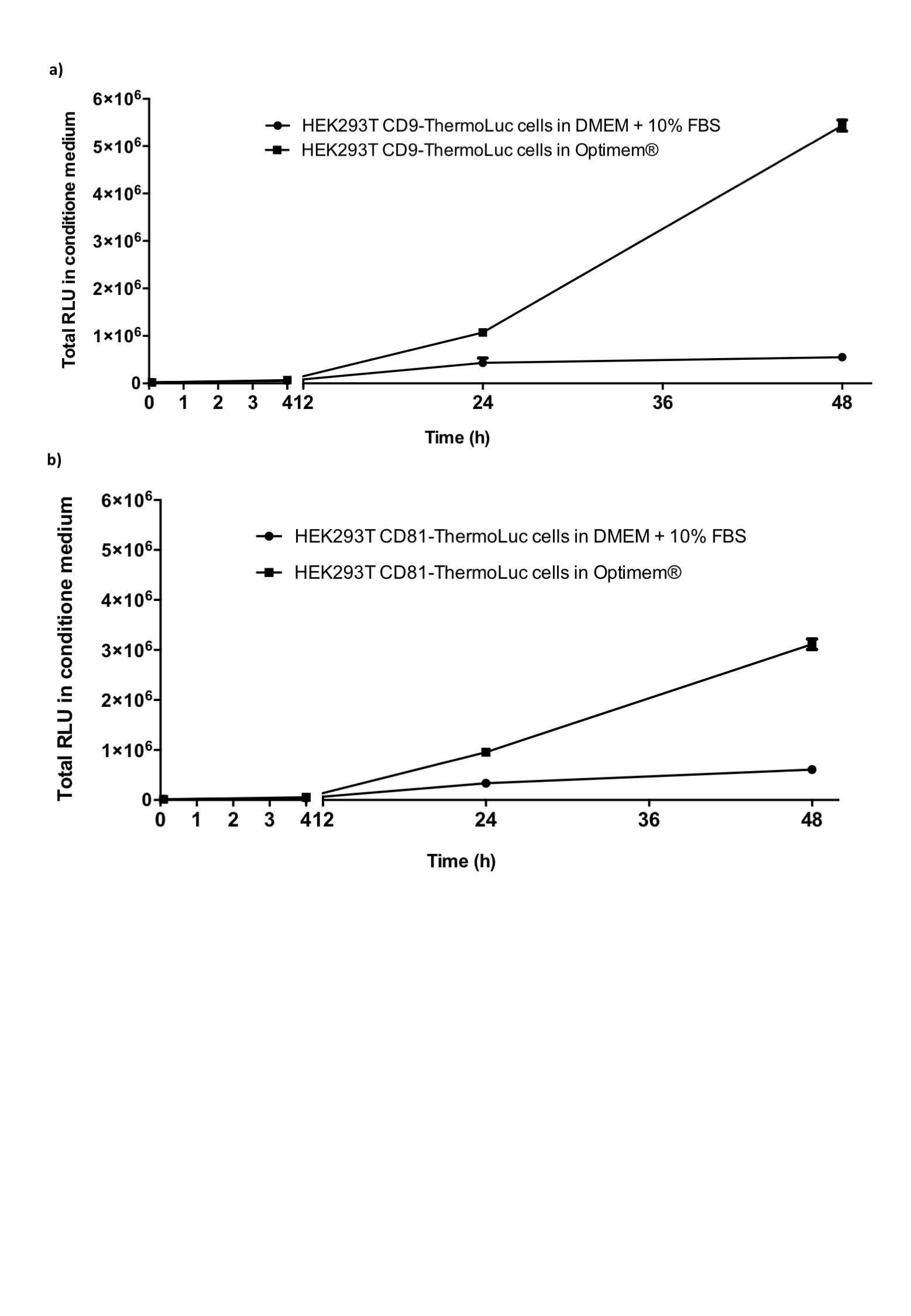

Supplement: Supplemental Material [file ZJEV_A_1800222_SM6146.zip › Supplementary/Supplementary/Supplementary Figure 8.tiff]

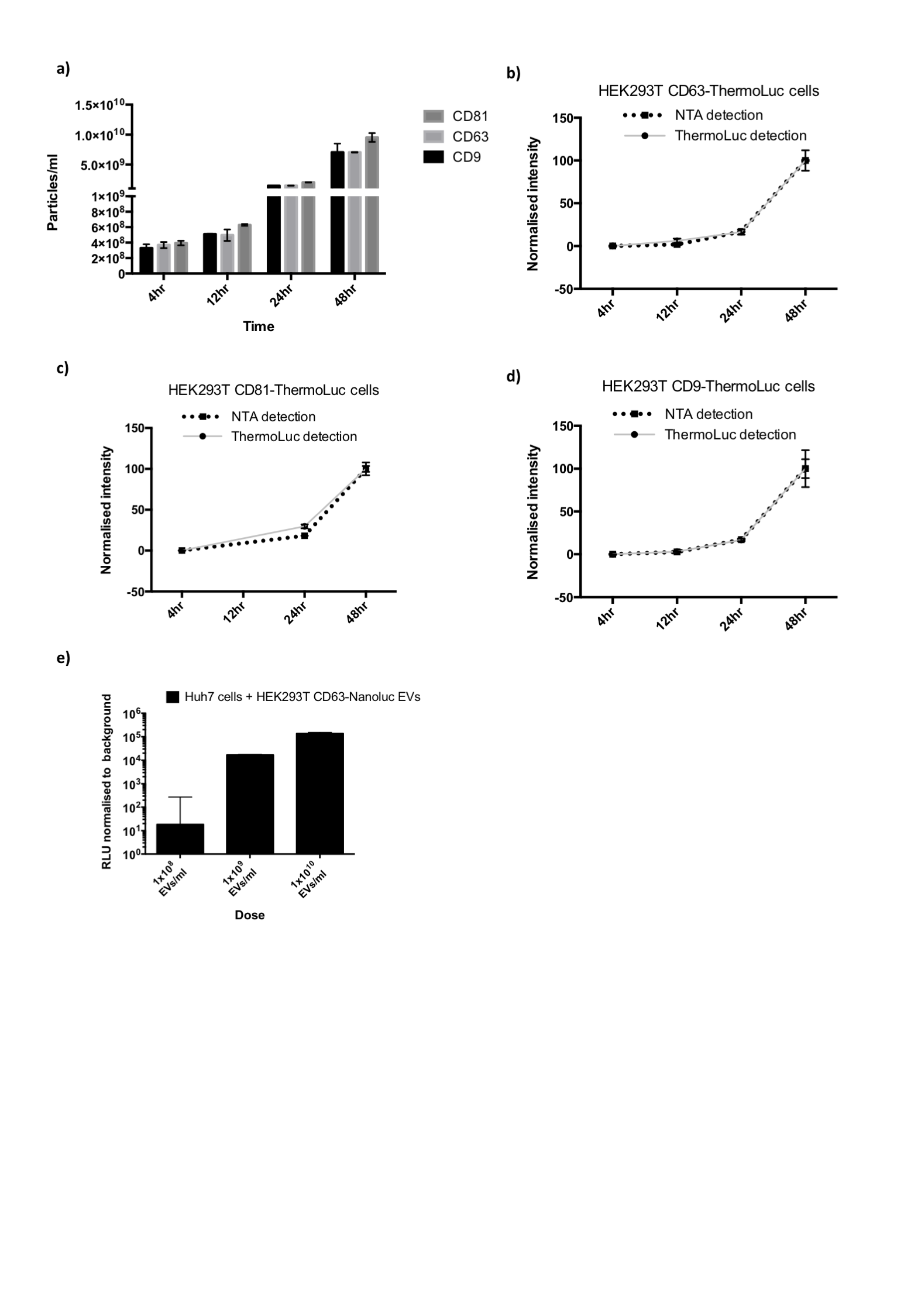

Supplement: Supplemental Material [file ZJEV_A_1800222_SM6146.zip › Supplementary/Supplementary/Supplementary Figure 9.tiff]

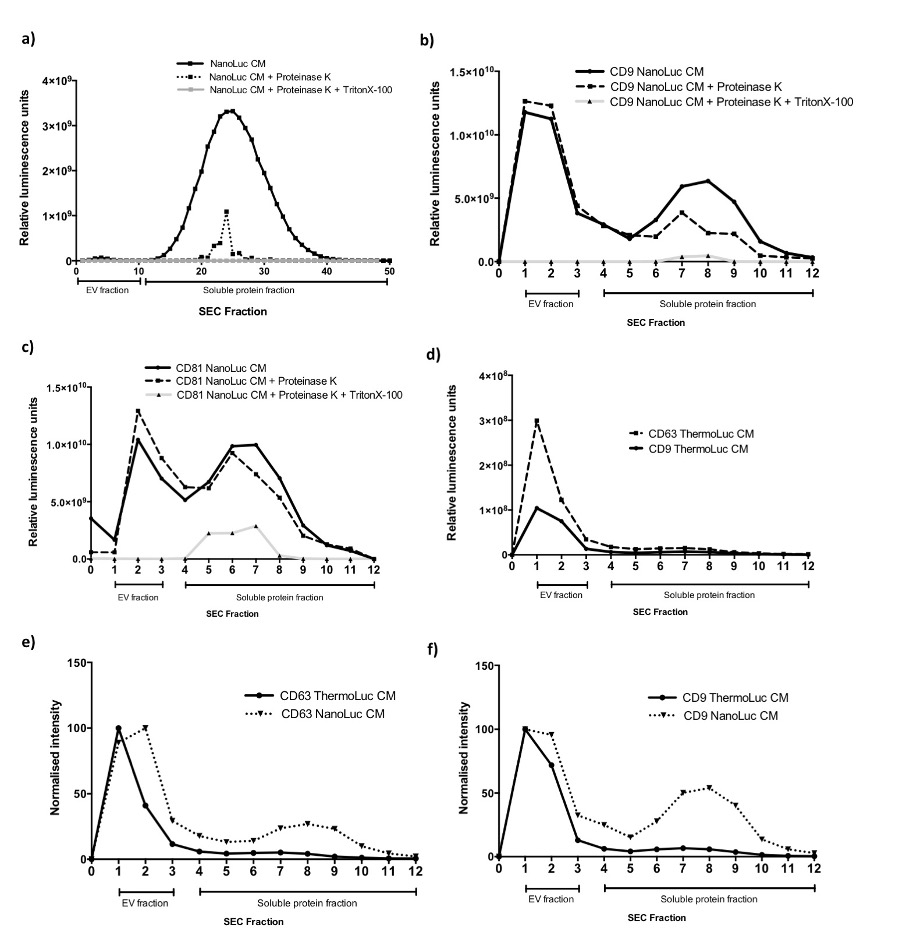

Supplement: Supplemental Material [file ZJEV_A_1800222_SM6146.zip › Supplementary/Supplementary/Supp_Figure_7.jpg]
